# *Yang*, forest animism, and environmental perception among the Bahnar in the Central Highlands of Vietnam

**DOI:** 10.1371/journal.pone.0354453

**Published:** 2026-08-26

**Authors:** Hoài Trần, K. David Harrison, Phước Lâm Huy Trần, Sơn Thanh Khuất

**Affiliations:** 1 UNESCO Chair in Environmental Leadership, Cultural Heritage, and Biodiversity, VinUniversity, Hanoi, Vietnam; 2 School of Interdisciplinary Sciences and Arts, Vietnam National University, Hanoi, Vietnam; 3 Center for Plants, People, and Culture, New York Botanical Garden, Bronx, New York, United States of America; 4 College of Business and Management, VinUniversity, Hanoi, Vietnam; Universitas Prima Indonesia, INDONESIA

## Abstract

In this paper, we explore Animism—Knowledge of nature spirits, and reciprocal relations among humans and non-humans—as a framework that the Bahnar people of Vietnam use for thinking about and interacting with their forest environment. Our case study is based on extended fieldwork among and consultation with the Bahnar people of the Central Highlands, who weave reciprocity with the spirit world into daily activities such as architecture, foodways, forest wayfinding, gong playing, plant gathering, agriculture, and river navigation. Understanding about the spirit world’s relations to the local environment and people is encoded in the Bahnar language through the lexicon—especially the emic concept *yang* which denotes spirits or deities—and is transmitted via metaphors, wise sayings, songs, stories, placenames, gong music, and epic tales by people who occupy diverse social roles. We hypothesize that the Bahnar human-spirit-environment cosmology is the primary lens through which they perceive nature and guides their daily interactions with it. We consider how their ancestral cosmology may additionally position the Bahnar as stewards of biodiversity and practitioners of sustainable living.

## 1. Introduction

We begin (Section 1) with a discussion of the location, history, and characteristics of the Bahnar ethnic group. We then turn (Sections 2, 2.1) to a brief review of how animism has been described and theorized in the literature, spanning diverse global regions from Amazonia to Siberia, with special attention to case studies from southeast Asia. We review recent Vietnamese language scholarship to understand how animism has been described and theorized in Vietnam by Vietnamese scholars. We then explore—based on our fieldwork—traditional animist views specific to the Bahnar people regarding the spirit world’s relationship with the local environment and its inhabitants. We will illustrate how Bahnar animism is embedded in everyday practices such as architecture, foodways, forest wayfinding, gong playing, medicinal plant gathering, river navigation, and rice agriculture. Animism is reflected in the Bahnar language through the lexicon, metaphors, wise sayings, songs, stories, placenames, and epic tales. We hypothesize that the Bahnar’s human-spirit-environment cosmology is a lens through which they perceive nature and guides their daily interactions with it. This ancestral cosmology, we argue, reflects the Bahnar view of themselves as stewards of biodiversity and practitioners of sustainable living. Our goal is to document aspects of Bahnar animism in both historical context and current practice, and to bring these into dialogue with global studies that connect animism and environmentalism.

The Bahnar are a prominent ethnic group in Tây Nguyên, the Central Highlands of Vietnam. They speak languages from the Bahnaric group within the Mon-Khmer (Austroasiatic) language family [1]. Primarily residing in Kon Tum and Gia Lai provinces, the Bahnar population was around 300,000 (in 2019) [2], p. 160, making them the largest Mon-Khmer speaking group in the region [3], p. 13 Most Bahnar people are bilingual, speaking both Bahnar and Vietnamese, and also understand Jarai.

**Fig 1:**
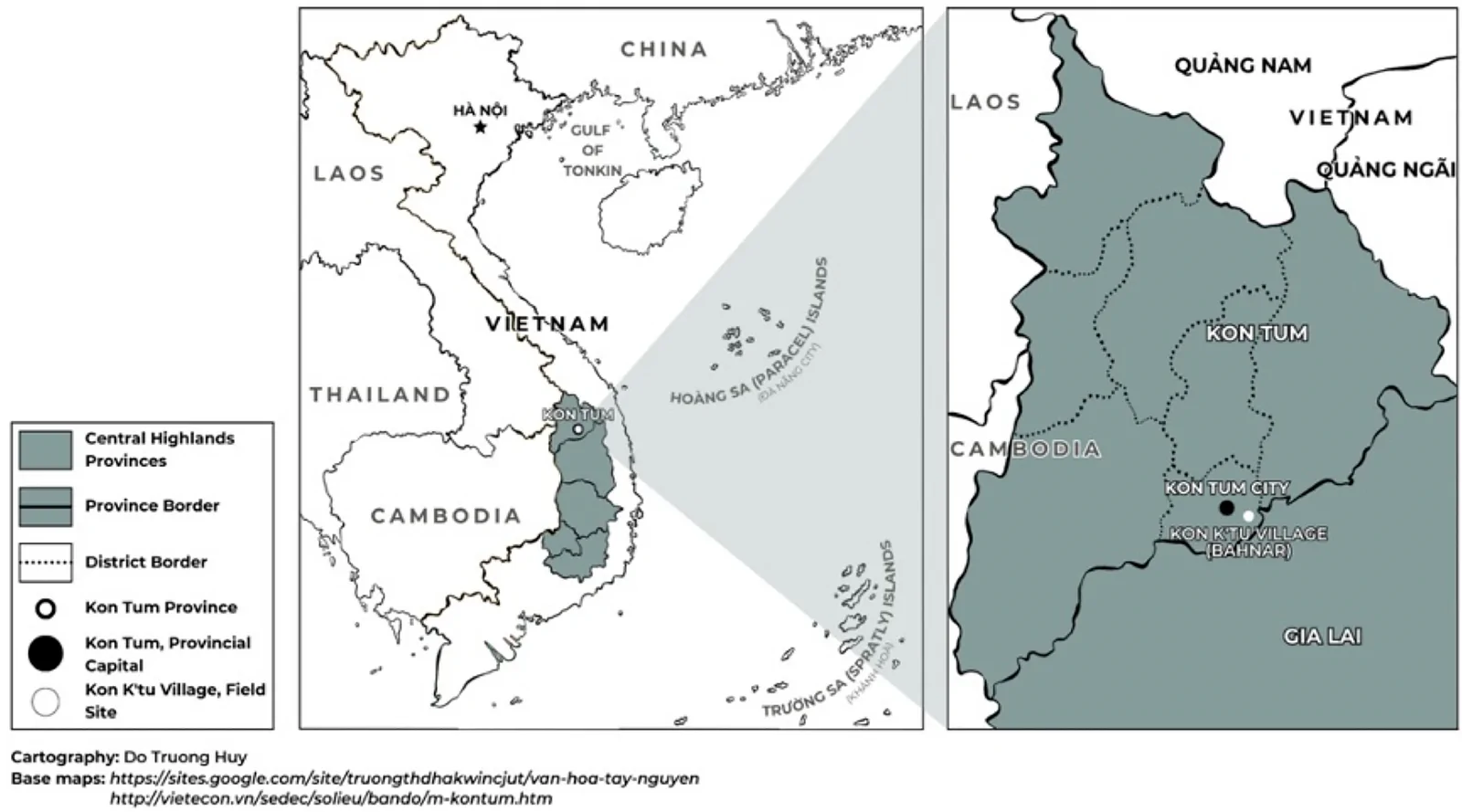
Map of field site at Kon K’tu village in central Vietnam. Map published under the CC BY 4.0 license.

Historically, each Bahnar village functioned as an independent entity led by a traditional chief (*bok kara*). The Central Highlands remained primarily isolated from lowland Vietnamese influence until the mid-19th century. In 1848, Vietnamese missionaries and French Catholic priests established a presence in Kon Tum Province, opening the region to outsiders. During the French colonial period, the French implemented policies to keep the Central Highlands and its Indigenous populations separate from lowland Vietnam, maintaining a strategy of colonial minorities (Fig 1) [4], p. 263.

Following the end of French colonial rule in 1954, the highlanders became involved in the Second Indochina War (1954–1975), which ended in 1975 with the Northern Communists emerging victorious. Subsequently, the Central Highlands were incorporated into the newly unified nation of Vietnam. After 1975, there was the integration of the Central Highlands into the broader Vietnamese state which brought significant changes. Government policies have aimed at economic development and cultural integration, sometimes leading to tensions over land rights and cultural preservation. The Bahnar people’s relationship with their environment has been significantly impacted by the ongoing political, economic, and social changes, resulting in a weaker connection as compared to the late 19th century and earlier periods. However, these challenges necessitate a thorough examination of how their environmental connection is maintained, particularly through the Bahnar’s human-spirit-environment cosmology that remains embedded in their daily lives.

## 2. Literature review

### 2.1. Apprehending animism

Animism as a concept has evolved greatly since first formally defined by Tylor [5], whose work drew on comparative reports from Indigenous societies in the Americas, Africa, Asia, and Oceania rather than first-hand experience. Tylor defined animism as the “belief in spiritual beings,” treating it primarily as an early stage in the evolution of religion in which humans attributed souls or spirits to animals, plants, and natural phenomena. In Tylor’s framework, animism is presented mainly as a system of ideas held by individuals about the supernatural world.

Anthropological research on Animism in the 20^th^ century, based on long-term ethnographic fieldwork, shifted the focus from belief to lived relationships. Humphrey and Onon, working among Daur Mongol communities in Inner Mongolia (northeastern China), describe a world where all manner of beings (not just humans) “articulate with their own energy and force” [6]. They describe Daur animism as not merely a set of beliefs but as social relations between humans and non-human beings (e.g., landscapes, horses), who are understood as agents with intentions, powers, and social roles. These relationships take place through everyday practices such as offerings at sacred *oboo* cairns, ritual burning of juniper and sprinkling of milk to local spirits, and respectful conduct such as not polluting water sources. As Humphrey explains: “landscapes are more in the nature of practices designed to have results: it is not contemplation of the land (*gazar*) that is important but interaction with it, as something with energies far greater than the human…[h]uman choice and agencies…are conceived as interrelated with and subordinate to the agencies attributed to entities in the land” [7].

Similarly, Vitebsky, based on fieldwork among Eveny reindeer herders in northeastern Siberia (especially Sakha Republic) describes animism as a system linking humans and non-humans via reciprocal communication (“dialogues”), mutual dependence, and shared social space [8]. Vitebsky’s work among the Sora in Odisha State, India, emphasized that Sora ancestral spirits (which remain active members of their families and villages after death), animal spirits, landscape spirits and other kinds of spirits are encountered as participants in daily life rather than as abstract objects of belief, and that animism emerges through lived engagement with them [9,10].

Anthropologists working in diverse locations have elaborated the relational approach. Bird-David, drawing on fieldwork among the Nayaka scheduled tribe of Tamil Nadu, India, argues that animism is better understood as a “relational epistemology”, not a mistaken belief about reality but a way of knowing the world that focuses on relatedness between beings [11]. Viveiros de Castro, working with the Araweté people in the Brazilian Amazon, described how their animistic ontology treats animals and spirits as persons with subjectivity and agency. He defines this view as follows: “Perspectivism is the conception according to which the world is inhabited by different sorts of subjects or persons, human and non-human, which apprehend reality from distinct points of view” [12]. Ingold, whose work draws on field research among Skolt Sámi reindeer herders in Finland, writes: “Animism is not a belief in spirits inhabiting a world of inert matter but is rather the experience of a world in which all beings are potentially animate and communicative” [13]. Ingold further observes, that from the Skolt Sámi point of view: “We do not live in a world of objects but in a world of beings with whom we share our lives” [13]. Descola, based on fieldwork among Achuar communities in the Ecuadorian Amazon, proposes a general model by which: “Animism imputes to nonhumans an interiority identical to that of humans while recognizing that their physicality differs”, where “interiority” denotes “the properties commonly associated with what we call the mind, soul, or consciousness” [14].

Within this rich scholarly tradition of defining and theorizing animism based on empirical case studies from regions outside Southeast Asia, we see in Bahnar animism both similarities and differences with other attested systems. In Descola’s concept of interiority, we see alignment with the concept of *yang* (a term which has been variously translated into English as ‘god’, ‘spirit’, ‘deity’, etc. and is explicated later in this paper) as documented among the Bahnar, Jarai, Katu, and Mnong peoples in Vietnam’s Central Highlands. In all four cases, a defining feature of *yang* is that these beings possess subjectivity, intentionality, and agency, and they participate in social relationships with humans. Bahnar grammatical structures encode *yang* as subjects of intentional action, recipients of offerings, and addresees in ritual speech. This linguistic evidence demonstrates that *yang* are conceptualized as person-like beings possessing subjectivity and agency, corresponding to what Descola terms “interiority”.

However defined in the literature, animism can also serve those communities that espouse it as a practical guide for human behaviors and adaptations in a changing environment. As many scholars have observed, people who hold to animist cosmologies have complex traditions of making offerings to nature (spirits), and rules or taboos that guide their behaviors with respect to animals, plants, and landscapes [15]. The Bahnar ethos of caring for the forest, for example, by talking quietly within it, attending to birdsong omens, and gathering only what is absolutely needed, aligns with Humphrey and Onon’s description of Duar animism as a form of caring for the landscape by respecting its resident spirits [6]. Similarly, Vitebsky describes Siberian Eveny animism as involving careful attention, respect, and moral responsibility toward the landscape and its spirit-owners. For the Eveny, who practice reindeer herding on the tundra, landscapes are understood as inhabited by spirit “owners,” and thus requiring respectful conduct toward land and animals [8]. Continuing the theme of animism as caring for landscapes, Peemot describes Tyva pastoralists’ “understanding of plants as respected beings which communicate between sentient superordinate homelands and human-nonhuman communities…plants’ special status ‘implie[s] respect towards them and regulation of the ways how people harvest and use them” [15]. Nadasdy, based on his work among the Southern Tutchone First Nation in Yukon Territory, Canada, observes how: ‘[l]ands are perceived as living entities, and they are active in relation to humans and animals. Lands nurture and sustain human beings. They are “intelligent actors in their own right” [16], p. 30.

As in these northern contexts, Bahnar interactions with forests, rivers, animals, landscape features, and spirit entities (*yang*) are structured through reciprocal social relationships grounded in respect, responsibility, and continuous engagement. These relationships are manifested in daily subsistence practices, in animal sacrifice rituals, and in traditional knowledge including taboo, and they reflect Bahnar lived social reality more than what had historically been described as a system of belief. Contemporary anthropology generally avoids “belief” because it is an external analytic category implying subjective conviction, and prefers, as we do, “knowledge,” understood as experiential and relational engagement with the world. In Bahnar society, spirits are not objects of belief but components of lived environmental knowledge. For these reasons, the relational concept of landscape-based animism articulated by Humphrey and Onon—and closely paralleled in Siberian cultures as described by Vitebsky, and by Peemot—provides a useful context for understanding Bahnar animism as a system of social relations between humans and non-human persons situated within a shared environmental and cultural landscape. We also agree with Stanford and Jong’s analysis, in the context of folk beliefs in Myamnar, that “…it might be argued that animism is not religion at all, but instead a form of spirituality” [17] These diverse manifestations and definitions of animism across many cultural settings provide a starting point for our discussion. We acknowledge—and with this paper hope to contribute to—ongoing discussions of how animistic worldviews inform stewardship of nature [18].

### 2.2. Defining *yang* as an emic category

The Jarai, Mnong, and Katu peoples inhabit the mountainous forest belt of central Vietnam, forming a continuous cultural zone with the Bahnar arranged in a north–south sequence along the Annamite Cordillera (Trường Sơn Range). The Katu occupy the northernmost portion, the Bahnar the north-central Highlands, the Jarai the central Highlands, and the Mnong the southern Highlands. These four cultures all possess an emic category *yang*, which we explicate here.

Among the Jarai and Mnong of the Central Highlands of Vietnam, *yang* refers to non-human persons inhabiting the forest and adjacent domains, a concept carefully documented and interpreted by both Dournes and Condominas. Dournes emphasizes that *yang* are not abstract beings but inhabitants of the physical world: “Le monde est peuplé de Yang” (“The world is populated by Yang”); while noting their geographic localization: “La forêt, la rivière, la montagne ont leurs Yang” [19], pp. 50, 56 (“The forest, the river, and the mountain have their Yang”). He describes *yang* as having individuated territorial presence: “Chaque lieu possède son Yang” (“Each place possesses its own Yang”) [19], p. 57. For the Jarai, Dournes asserts, the forest is not empty space but a socially populated domain inhabited by *yang*, which he sometimes poetically describes in French as “les fééries de la forêt” [20], which may be translated as “the fairy-like spirit presences of the forest,” conveying the experiential sense of the forest as animated by a multiplicity of invisible, agentive beings.

Condominas similarly describes the Mnong understanding of *yang* (which he translates variously as *genies* in French and ‘spirit-beings’ in English) as territorial presences inhabiting specific parts of the forest [21]. Referring to the powerful territorial spirit at his Mnong field site, he writes: “la pierre-génie Gôo” [22], p. 12, p. 23 (“the stone-spirit Gôo”), identifying a *yang* that inhabits a specific stone formation in the forest. He further explains the spatial distribution of *yang*: “Chaque élément du paysage possède son génie propre” [22], p. 45 (“Each element of the landscape possesses its own spirit”), and emphasizes their pervasive presence in the forest environment: “La forêt est pleine de ces génies invisibles” [22], p. 62 (“The forest is full of these invisible spirits”). Together, Dournes and Condominas show that *yang* are understood by the Jarai and Mnong not as abstract deities but as socially interactive, territorially located non-human persons inhabiting the forests, rivers, mountains, and other domains, and are bound up in mutual relationships with human communities.

Århem conducted his Katu fieldwork in A Lưới District, Thừa Thiên Huế Province, located in the northern Annamite Mountains approximately 150–250 km north of Bahnar territory in Kon Tum and Gia Lai provinces. The Katu and Bahnar occupy contiguous territories along the Annamite mountain chain, sharing closely related ecological environments, subsistence systems, and animist cosmologies. Århem distinguishes two major categories of Katu spirits using the indigenous terms *abhuy* and *yang*, which together populate what he describes as a “spiritually alive – a sacred – landscape” requiring constant interaction and negotiation [23], p. 5. The broader Katu category *abhuy* refers to spirits inhabiting the landscape, including forest, land, and water spirits; he explains that “the Katu…use the single word *abhuy* to denominate all of these entities and do not regard the spirits of dead people as fundamentally different from the spirits dwelling in the landscape” [23], p. 81. These spirits are ubiquitous and territorial, since “each hill and patch of forest may have its own legion of *abhuy krung* (forest spirits)” [23], p. 95, and they actively influence human life, requiring rituals, offerings, and respectful conduct. By contrast, the Katu term *yang* refers specifically to the spirits of deceased ancestors who dwell in the household and act as intermediaries with other spirits; in agricultural rituals, Katu villagers “primarily address their ancestors (*yang dong*; literally ‘house spirits’), asking them to act as their representatives and to deal, on the family’s behalf, with the spirits of the swidden field (*abhuy hare*)” [23], pp. 89–90. Thus, while *abhuy* is the general term for Katu landscape spirits—sometimes rendered by Århem as “ghosts”—*yang* denotes a restricted category of Katu ancestor spirits residing in the house and maintaining ongoing social relations with the living.

### 2.3. Animism from Vietnamese scholars’ perspectives

There is a knowledge gap about animism in Vietnam, because much of the scholarly literature is available only in Vietnamese. To bridge the gap, we reviewed more than 40 academic published papers related to animism as practiced by Vietnam’s ethnic minority peoples. The papers we reviewed were published within the last decade (2004–2024) in Vietnamese academic journals, including *Tạp Chí Văn Hoá Dân Gian* [Journal of Folk Culture], *Tạp chí Khoa học xã hội miền Trung* [Central Vietnam Review of Social Sciences], and *Tạp Chí Di Sản Văn Hoá* [Journal of Cultural Heritage]. The reviewed papers cover seventeen ethnic groups mostly inhabiting Northwest Vietnam and Central Highland Vietnam including: Bru, Hre, Ede, Bahnar, Jarai, Thai, Tay, Katu, Nung, Muong, Si La, Hmong, Phu La, Ro Man, San Chi, Cham and Xo Teng. In Vietnamese scholarly writing, the term indigenous (*bản địa*) is not used, because of the official state policies which prefer the term ‘ethnic minority’ (*dân tộc thiểu số*) or ‘local community’ (*dân tộc tại chỗ*). Despite the avoidance by scholars of the label indigenous, ethnic minorities of Vietnam are commonly regarded as the original inhabitants of their ancient ancestral homelands.

Vietnamese anthropologists have described animism as *Vạn vật hữu linh* (‘everything has its spirit’), but the term needs to be understood in a local perspective. Recent literature in Vietnamese investigates the characteristics and practice of animism across the span of daily activities among ethnic minorities; in illness treatment among the Thai people [24]; agricultural activities of the Ede, Tay, and Nung peoples [25–27]; the *Then* dance of the Tay people [28]; and the funeral rites of the Ede people [29]. According to Thủy, the Bahnar “believe that everything around them has the existence of gods (*yang*), of supernatural spirits that govern them and regulate their thoughts and actions” [30]. These studies by Vietnamese scholars resonate to some degree, but in other ways diverge from the literature on animism in Vietnam and in diverse cultures outside of SE Asia. Collectively, the works reviewed here reveal significant local differences in animistic practice, with some trans-regional or global patterns.

All the above works reflect the tight coexistence between people and nature spirits which, in Vietnamese ethnic groups’ beliefs, reside in natural elements. To show respect for the forest and mountain god, the Muong people conduct forest closing and opening ceremonies during the lunar new year celebration days. Within this period, villagers are not allowed to enter the forest for hunting until after the forest opening ceremonies [31]. In another case, the Ede people believe in the Rice god (*yang Mdie*) as a living being. During the early harvesting season, the Ede use their bare hands, instead of sickles, to collect rice in order not to hurt the Rice God [29]. Comparable human-spirit relationships can be seen in the *yiang* of the Bru people [32], the *Sinh Ca* folksongs of the San Chi people [33], or the buffalo-eating ceremony of the Xơ Teng people [34].

However, most of the Vietnamese scholarly literature we reviewed also positions ethnic minorities and their animistic beliefs in what Hoàng Cầm describes as a ‘state of disintegration of primitive society’ (*phạm trù tan rã của xã hội nguyên thủy*) [35]. When commenting on animism, Vietnamese researchers often use phrases like ‘fading away’, ‘superstition’, ‘taboo’ or ‘irrelevant to new life’. In reference to that, Trần [36] has concluded that Vietnamese state ethnographers’ work has to be ideologically filtered to ensure its alignment with the Party’s policies toward minority groups. Within this socialist discourse filter, the cultural practices of the minorities are deemed to be primitive and superstitious, thus causing poverty and social stagnation. There is no discussion by these authors of how Bahnar animist beliefs and views might contribute positively to environmental care.

In the case of the Bahnar, in 1937, Nguyễn Kinh Chi and Nguyễn Đổng Chi contributed one of the earliest and most voluminous ethnographies [37]. In their work, the Nguyễn brothers describe Bahnar culture in detail, including population, customs, social structure, tangible culture (houses, baskets, and textiles), cuisine, and many other aspects. One crucial part of the work is the authors’ respectful description of traditional beliefs among the Bahnar people. As Nguyễn and Nguyễn report, the Bahnar have a system of mystical beliefs that: ‘anyone with a bit of scientific thought could not help but laugh when they heard it’ [37], p. 41. However, Nguyễn and Nguyễn also insist based on their careful investigations that, for the Bahnar, animist beliefs are so important as to constitute the ‘driving force’ and ‘the foundation of their daily life.’ Thus, the ethnographers emphasize that ‘if you want to understand the interesting customs of the Bahnar people, it is best to first know about their (animist) beliefs’ [37], p. 41. In this spirit, our research describes Bahnar animism as a dynamic and adaptive mode of thinking and acting that spiritually connects individuals to their surrounding environment. Animist beliefs, as we will demonstrate, emerge not merely as a singular element of folk culture but through active and ongoing interaction by human beings with their environment.

### 2.4. Animism in Southeast Asian context

Beyond the Vietnamese Central Highlands, forest animism is attested more broadly across Southeast Asia among swidden agriculturalists who also hunt, gather, and manage forests and among hunter-gatherers. The Jarai of Cambodia, according to Padwe [38] “recognise the landscape as animated and potent”, and their cosmology “emphasiz[es] the agency of the natural world.” Among the Chewong of Peninsular Malaysia, Howell emphasizes that non-human beings inhabiting the forest are understood as persons sharing the same ontological status as humans: “Animals, plants, and spirits are persons” [39], p. 148, and she further explains that “the forest is inhabited by spirit-beings who must be treated with respect” [39], p. 67, highlighting the forest as a socially structured environment populated by agentive, non-human persons. Similarly, Endicott describes the Batek Negrito cosmology in which spirit-beings actively inhabit and regulate the forest world, writing that “superhuman beings control the processes of nature” and emphasizing that: “The Batek believe that superhuman beings exist and influence their lives” [40], p. 18. Working among the Phunoy people of Laos, High writes: “The land is not empty but inhabited by *phi*, spirits who must be respected and propitiated” [41], p. 156. She further observes that for the Phunoy: “Entering the forest without proper ritual acknowledgement risks offending the *phi* who reside there” [41], p. 158. In these three cases, as we have observed among the Bahnar in the Central Highlands of Vietnam, forest environments are not seen as inert spaces but as socially and morally structured domains inhabited by agentive non-human persons, with whom humans must carefully maintain ongoing relationships of respect, reciprocity, and interdependence.

## 3. Methods

This section outlines the qualitative ethnographic methods used in this study, including participant recruitment, participant observation, note-taking, data elicitation, coding, and analysis, as well as considerations of researcher positionality and analytical validity. Methods included systematic review of recent literature, participant observation, semi-structured interviews, audio and video recording, photography, linguistic corpus analysis, digital lexicography, and co-authorship with local experts. A research team of three Vietnamese scholars and one American researcher conducted eight field expeditions to Bahnar communities between 2022 and 2024. Participants were identified through referral sampling, and cultural data were documented through field notes, photographs, and audio-visual recordings with community participation and informed consent. All individuals named or shown in this manuscript have given written informed consent (as outlined in PLOS consent form) to publish these case details. The individuals pictured in Figs 3 and 4 have provided written informed consent (as outlined in PLOS consent form) to publish their image alongside the manuscript. Copies of collected materials were repatriated to the community and archived (https://doi.org/10.5281/zenodo.15837888). The study was approved by the Institutional Ethical Review Board of Vinmec International General Hospital (Decision No. 77/2021/QD-VINMEC, March 20, 2021). Our secondary methodology was analysis of legacy dictionaries; a rich source of spiritual-environmental knowledge [42]. This paper thus draws upon three sources: the recent Anthropology literature (mostly in Vietnamese), first-hand accounts collected from the Bahnar community, and historic lexicographic materials. Together, these provide a comprehensive and holistic view of Bahnar animism.

### 3.1. Lexicographic methods and replicability

We fully digitized two bilingual dictionaries, Bahnar-English [43] and Bahnar-French [44]. These contain ca. 3,000 headwords and ca. 5,000 headwords, respectively, as well as example sentences. While even a collection of 8,000 lexemes cannot be considered as complete or exhaustive, we believe this is the most thorough sample of Bahnar vocabulary that has been compiled by anyone. We also consulted a second Bahnar-French dictionary [45], but did not digitize it due to the complexity of the data structure. However, we used it to cross-check and look up words found in the other two dictionaries. We kept the two digitized dictionaries separate, each one comprising a searchable linguistic corpus. We used targeted searches to find all dictionary entries and definitions relating to animism (e.g., god, heal(er), magic, spirit, sacred, ritual, sacrifice). We also generated thematic word lists comprising animal names, canoe-related terms, landscape terms, plant names, time-related terms, verbs relating to environmental activities, etc.). We read both dictionaries in their entirety to find terms that we might have missed during our keyword searches. The result of this was a list of thematic vocabulary relating to Animism (ca. 250 terms). We then recruited a trilingual native speaker (Bahnar-Vietnamese-English) validated the pronunciation and meaning of every word appearing in thematic lists. He assisted us to make native-speaker audio recordings of those words for the Bahnar-English Talking Dictionary. Each lexical token was recorded three times to ensure quality. Our consultant was able to confirm all but a few terms. Any terms that he identified as unfamiliar or unknown to him could still be cited by us as historical/dictionary forms, even though they may not be in current usage. We also participated in a wide range of environment-related activities, for example constructing and erecting the sacred *gâng* pole, harvesting rice, foraging for useful plants and edible insects, pond-field preparation, cooking food, canoeing on the river, collecting plants in the forest. During and after all these activities, we had conversations with our Bahnar experts about the activities and took field notes.

### 3.2. Subjectivity

As ethnographers, we recognize our own subjectivity and positionality and acknowledge how as non-Indigenous, male researchers our personal backgrounds, experiences, beliefs, and social identities may influence our observations, interpretations, and interactions during fieldwork. Accordingly, we incorporate the following practices: (a) reflexive approaches to make the interpretive process more transparent; (b) peer debriefing with colleagues; (c) attention to power dynamics related to the socio-political status of ethnic minority communities in Vietnam and within our research team, which includes Vietnamese (authors #1, 3, 4) and American (author #2) scholars at different stages of career seniority; (d) inclusion of positionality statements in fieldnotes and public presentations of data; and (e) sharing analyses and draft manuscripts with community consultants to ensure accuracy and avoid misinterpretation.

### 3.3. Participant recruitment

We adopt a general approach of knowledge co-production, rather than data extraction. As such, we fully acknowledge by name all our Banhar cultural experts who shared their knowledge and include them as named co-authors on published work whenever possible. We repatriate copies of all our recordings to the community, recognizing that the recordings are their intellectual property. To explore the theme of Bahnar animism, we obtained permission from local authorities to work in the Bahnar community. We submitted our project to the Ethics Review Board at VinUniversity and received a waiver for human participants research (see supplementary material). Further, we obtained permission from the leadership (e.g., current and former chiefs) of the Bahnar community. Upon commencing fieldwork, we recruited 20 expert consultants. We identified these Bahnar experts by referral and purposive sampling. Additional information regarding the ethical, cultural, and scientific considerations specific to inclusivity in global research is included in the Supporting Information (S1 Checklist).

### 3.4. Participant observation

One author lived in the Bahnar community for an extended period as a participant observer from July 2016 to June 2017 (11 months) and then two more months (June-July 2018). All authors participated in two or more of the eight short-term visits we made to the community, lasting up to 5 days each, in 2022–2024. We hosted a reciprocal research visit by bringing 3 Bahnar culture experts to Hanoi in 2024. During field visits, we participated in many diverse activities including harvesting rice, navigating canoes on the Dak Bla river, foraging for insects, collecting useful plants, making handicrafts, preparing traditional foods, learning and singing Bahnar songs, playing gongs, weaving baskets and textiles, carving wood, participating in certain rituals, preparing and drinking rice wine, preparing and sowing pond fields, gardening, building vernacular architecture, walking in the forest, playing sports in the village, etc. Our participation in such activities fostered a naturalistic setting for casual conversations on many topics, which provided us with a great deal of cultural knowledge and insights, as well as some hands-on skills.

### 3.5. Fieldnotes

We took daily notes/diary entries during our time in the community, which resulted in more than 200 pages of notes, in Vietnamese, Bahnar or English. Fieldnotes were taken in accordance with best practices in anthropology, which continue to evolve, and provide a foundation for ethnographic work like ours [46,47]. All notes are stored in our project archives at our home university, and available to be shared with researchers upon request. In the supplementary section of this paper, we include sample pages of our field notes. Our detailed field reports are archived at Zenodo [48]. For any researcher seeking to replicate our study, we recommend this engaged model of participant observation and qualitative ethnographic analysis.

### 3.6. Qualitative data collection

We conducted approx. 100 semi-structured interviews (rather, guided conversations, as we did not pre-formulate specific questions) during which we took field notes and/or made audio/video recordings. Interviews were conducted in Vietnamese, Bahnar, and English (in some cases with simultaneous interpretation). Recording was done with prior informed consent of the consultants, which we also recorded verbally. Handwritten field notes were taken mostly in Bahnar and Vietnamese, and to a lesser extent in English.

We took approximately 2,000 documentary photographs, several of which appear in this paper. All photos are in our project digital archives, and we deposited copies of selected photos (both digital and print) with the Bahnar community. All videos are kept in our project digital archives, and some, with permission of the participants, have been posted on our YouTube channel.

### 3.7. Qualitative analysis

Our analysis adopts a grounded theory approach, assuming that both the digitized dictionaries, as well as the unstructured or semi-structured verbal narratives provided by our consultants would, upon subsequent analysis, reveal emergent cultural patterns and important themes. We coded our notes and participant interview transcripts for all the above themes, plus material culture objects such as the sacred *gâng* pole, totemic bird carvings, textiles, culture heroes, vernacular skills (e.g., foraging for insects), and various folk beliefs. Bahnar culture is very rich, and some domains of knowledge are sensitive, proprietary, or secret. Thus, we are fully aware that we have not reached a point of theoretical saturation, defined as “…when no additional themes of insights emerge from the data collection, and all conceptual categories have been explored, identified, and completed” [49]. In fact, this is a receding horizon for the study of any culture, but it does not diminish the relevance of anthropological field studies. In the history of anthropology there are a few exemplary cases where the researcher has managed to collect and catalog every single material culture artifact (e.g., Harold Conklin’s collection of Hanunóo artefacts at the Yale Peabody Museum), but we are not aware of any study, no matter how thorough, that has claimed to have collected every cultural concept or every word of a language.

It is inevitable that certain Bahnar beliefs, concepts, artefacts, or practices have as yet eluded our attention, and so we consider our research to be preliminary and in progress. Nevertheless, based on our compiled lexical list pertaining to animism, we believe we have reached a point approximating theoretical saturation with respect to Bahnar mythical-spiritual entities (e.g., names of gods and spirits), and typology of rituals.

We sought to validate our data by comparison of distinct data sources (multiple consultants) and using different methods (interviews with observations). We checked for validity by looking for consistencies and inconsistencies in data reported by our consultants, and by seeking alternative descriptions and explanations. We used analytic induction and acknowledgment of negative evidence in order to generalize by means of inductive reasoning. We shared and discussed our data and tentative conclusions with community members for further validation, and to ensure that no sensitive or confidential material was included.

## 4. Language and environmental-spiritual knowledge

We adopt a Bahnar metaphor of language as a basket containing traditional knowledge [50]. The Bahnar animistic worldview is expressed across all Bahnar language genres. Our linguistic survey—which includes dictionaries written more than a century ago, orally transmitted tales, and present-day narratives—helps to uncover rich aspects of Bahnar animist cosmology expressed in the language.

### 4.1. The lexicon of Bahnar animism

A diachronic comparison of three key sources reveals a striking evolution in how *yang* has been translated and conceptualized by outsiders over more than a century. Three Bahnar dictionaries we surveyed provide a wealth of animistic terminology, including names for gods, spirits, mythical beings, rituals, and paraphernalia for communing with spirits. To check present-day usage, we also confirmed these terms with our Bahnar language consultants.

In his *Dictionnaire bahnar–français* (1889), the French Catholic missionary Pierre Dourisboure glosses *Iāng* (n.) as “spirit, divinity,” and he prioritizes usage examples in a Christian context: *Iāng Bă* “God the Father”, *Iāng Kon* “God the Son”; followed by traditional usages (which he marks as “Superstition”): *iāng zờn ‘spirit of the harvest’; iāng kông ‘spirits of the mountains’, iāng dak ‘spirits of the water’, and iāng teh ‘spirits of the earth’* (p. 133). His definition shows how Yang has been adapted to the context of Christianity while still retaining its meaning of spirit beings located in mountains, waters, and fields. These nature spirits are distinguished from human-assisting spirits: *gru, guru* (n.) ‘Spirit which is supposed to assist the pythonisse’ (clairvoyant, prophetess); and from ancestor spirits *kǐăk, kǐek* (n.) ‘A dead man, a cadaver. The souls of the dead, ghosts. Spirits, divinities’. Other mythical-spiritual entities mentioned include *bodruh* (n.) ‘Imaginary insect that causes dysentery and that the pythonisse makes the pretense of removing to cure the patient’, and *prao* (n.) ‘Dragon, monstrous serpent’. Human interactions with the spirit world may result in specific consequences, such as *xỏnoh* (n.) ‘Debt allegedly contracted towards spirits by the violation or omission of a superstitious practice. (One exposes oneself to death if one does not hasten to pay it.)’; and *kỏchít* (v.) ‘To make perish, to perish. (The pagans say: *Kǐěk kỏchít xỏ*, the spirits made him die…)’.

Seventy years later, Paul Guilleminet, a French colonial administrator and ethnographer, in his monumental *Dictionnaire bahnar–français* (1959) with A. Alberty, defined *yang* (spelled as *iang*) as: “Supernatural powers, mostly male, living ‘everywhere’ and especially in a ‘third world,’ but penetrating onto the earth—which is the first world—and among the Mânes (ancestral spirits), who live in a second world. The Yang ensure that humans observe the rites; through their faults, humans provoke anomalies and are then punished by the Yang.” For Guilleminet, Bahnar *yang* are situated within a layered cosmology and positioned as regulators of ritual propriety. Important divinities in Bahnar are given proper names, which may also include the lexeme *yang*. These include *Bok Glaih* (god of thunders), *Iang Xơri* (or *Iang Đai* – god of rice), *Iang Đak* (god of water), *Dak Iang Kong* (god of the mountain), and *Kong Iang Long* (gods of trees). There are also individual named tree gods, such as *Long Jơri*, *Long Hara*, *Long Breng*, *Long Kachit*, and *Long Kơtrac*. He further glosses *yang* as “sprits”, “genies”, or in one instance “clever genies, difficult to deceive”. He also notes the semantic extension of the term; among Bahnar who practice Catholicism he records that *Yang* has come to mean “God, Divinity, divine, sacred”.

Finally, the Protestant missionary linguists John and Elizabeth Banker, working with Bahnar consultant Mở under the auspices of the Summer Institute of Linguistics, defined *yang* simply as “spirits, nonhuman beings that affect humans,” thus abandoning theological and cosmological hierarchy in favor of a functional, interactional description. They also include various spirit entities: *hodrĕ* ‘An evil spirit’, *hri ~ yang hri* ‘The spirit that makes rice grow’, *gơhlŏu ~ gơhlâu* ‘An imaginary object that lives in trees, has good smell’, *hui hai ~ yă hui hai* ‘Name of imaginary people, amazons’, and *kiĕk wir* ‘A tiger that is able to make itself into a man’. Verbs associated with spirits include: *ming yang* (v.) ‘To placate the spirits, the term used when sacrifices are made’, *soi* (v.) ‘To commune with the spirits, sacrifice’, *sơnoh* (v.) ‘To pay to spirits what has been promised’, *tơl* (v.) ‘To answer, to promise the spirits a sacrifice’, *pơwir* (v.) ‘To cause the self to change into an animal or vice versa’, and *sơkat* (v.) ‘To speak with magical power, to speak and have something magical happen’. Spiritual accessories include *sơtôk* (n.) ‘Type of wine jar which is the most expensive and has a spirit associated with it’, *ư-ŏ-pơgâng ư-ŏ* (n.) ‘Type of medicine used to stop spirits from entering the village’, *sơlỗ* (n.) ‘A magic medicine that blinds the eyes of others for a while, and *thŭ* (n.) ‘Magical word used to make things happen’. A shaman, called *pơjâu* (n.) in Bahnar, may be said to *mơsêh* (v.) ‘have supernatural magic’ or *mơyang* (v.) ‘have supernatural powers’. Our Bahnar language consultants in Kon K’tu village confirmed that these terms are still known within the community, although they describe some practices now seen as obsolescent. Taken together, these three dictionaries provide a rich lexicographic record of the Bahnar animistic/environmental lexicon, illustrating a trajectory from missionary lexical equivalence (Dourisboure), through mid-century cosmological systematization (Guilleminet), toward relational and interactional formulations (Banker et al.) that implicitly dissolve the natural/supernatural divide and present *yang* as nonhuman agents embedded in lived ecological and social space.

### 4.2. Animism in Bahnar Epic Tales

**Fig 2:**
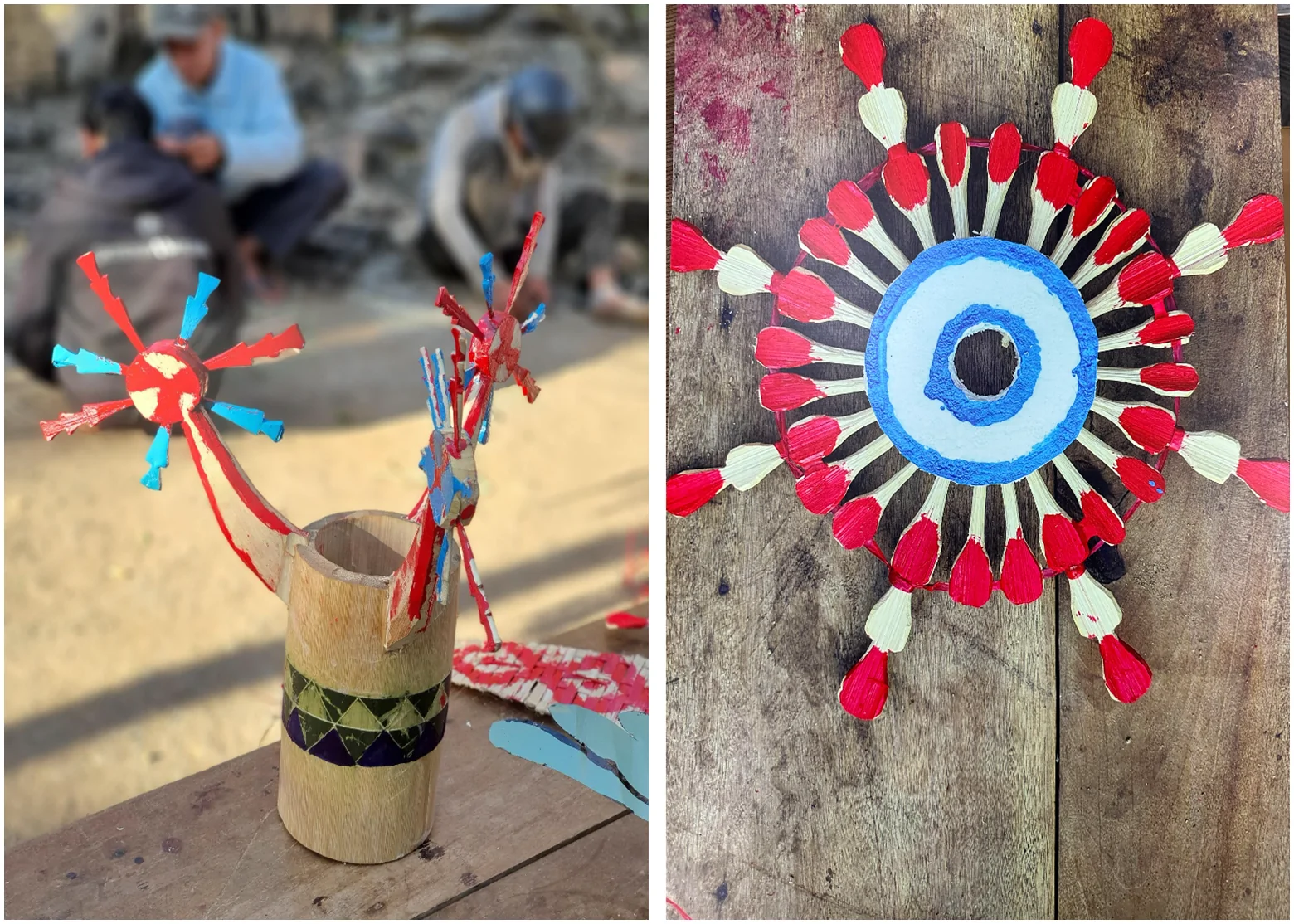
Symbols of the Sun (*Măt Nar*) as a decorative element of the Bahnar ritual *gâng* pole. Photos by the authors, published under the CC BY 4.0 license.

Bahnar environmental knowledge is passed on mostly by oral tradition, for example in epic tales called *hơmon*, which may be glossed as ‘old-time stories’. Even though the Bahnar epic’s focal narratives center on the exploits of heroic figures, they disclose an extensive societal context, encompassing material existence to spiritual dimensions and spanning from customs, festivals, and rituals to insights into geography, culture, and folk wisdom. We compiled a corpus of Bahnar epic tales [51,52] and then identified all spiritual and environment-related terms (S1 Appendix). The Bahnar epic tales describe close human interactions with *yang* and with the gods *Yă Kung Keh* and *Kok Kei Dei* as the highest traditional Bahnar deities who interact with people. A central theme and story in the Bahnar epic revolves around the actions of heroes whom good gods assist in fighting evil gods/spirits. For example, in the Bahnar epic Dam Noi, Dam Noi is the result of the marriage between the mortal Dam Set and the daughter of the god Bok Kei Dei – Bia Rak. With the power of half-god, half-human, and the help of the gods, Dam Noi defeats the evil gods and protects his village. Thus, in the eipc and mythological world of the Bahnar people and their relationship with the deities, humans are not always passive and weak but have certain impacts on the divine world. People connect, negotiate, and pray to *yang* through rituals (Fig 2). They can also fight against and defeat *yang* that may harm the villagers [51].

In the Bahnar epic tales, *Bok Kei Dei* and *Yă Kuh Keh* are deities who created all living things. Accordingly, *Yă Kuh Keh* made heaven and earth, and *Bok Kei Dei* made the sun, moon and stars. In the lower hierarchy are many other nature deities that govern human life, such as the Sun spirit (*yang Măt Na*), Moon spirit (*yang Măt Khei*), the female Rice spirit (*yang Sri*), the female Water spirit (*yang Dak*), the Fire spirit (*yang Unh*), the Forest spirit (*yang Bri*), the Earth spirit (*yang Teh*), the Rock spirit (*yang T’mo*), the Mountain spirit (*yang Kong*), or River spirit (*yang Krong*). Besides the good spirits, the Bahnar believe there are evil spirits who disturb people’s lives, such as *Bok Hoch* (a giant bird with a wide wingspan and a terrifying cry), in charge of wild birds and animals, often herding wild birds and animals during the ripening rice season, thus causing harm to people’s crops. The Bahnar people also fear the god of Thunder (*Bok Glaih*), a strong and majestic male deity that terrifies people [51].

According to Nguyễn [53], the relationship between the divine world and human characters in the Central Highlands epic is a two-way relationship of interactions that can be both contradictory and harmonious. For the Bahnar people in particular and other Central Highlands people in general, deities may be good and bad. Good deities often help and bless people’s lives, while bad ones specialize in harming human lives. The language of the Bahnar epics represents not only a genre of folklore but also a medium of animist expression and environmental knowledge. In the epics, Bahnar conceptualize deities of nature with human-like characters and describe them in close interactions with human beings.

## 5. Bahnar animism and the sacred

The Bahnar animist lexicon (S1 Appendix) aligns well with relational analyses of animism, in which beings emerge through their relationships with humans, each other, and the environment. In Bahnar cosmology, *yang* and other deities are personified and perceived as having human-like characteristics. The relations between the Bahnar and supernatural entities are negotiable and interactive via rituals and prayers, as the natural spiritual powers can be both scary and familiar, forceful and negotiable. Bahnar interactive and negotiating relations with their surrounding sacred environment is ongoing, and very much in line with this model. Furthermore, we find that Bahnar animism creates a mindset that encourages the community to protect and steward its natural environment [25].

### 5.1. Bahnar *yang*

In Bahnar cosmology, the *yang* are perceived as having human-like characteristics. Our above survey based on the Bahnar language, lexicon and epic tales related to animism indicates that naming the *yang* and recognizing their characters is a way of showing connections between nature and the spiritual world. According to McNamara et al. [54] on cognitive science approaches to religion, “the human ability to conceive of and perceive the divine comes from…how human minds conceptualize the mind(s) of god(s) as an extension of the ways we evolved to understand other human minds….” In Bahnar belief, each *yang* has particular human-like characteristics that aligns with their spiritual abilities and influences on people. Nguyễn and Nguyễn’s ethnography [37] describes the reciprocal relations between villagers and *yang.* In a comprehensive overview of Bahnar belief, based on works of previous scholars and her own observations, Thủy describes particular characteristics of two crucial *yang* in the beliefs of a Bahnar agricultural community: the Sun spirit *Yang Măt Nar* and the Moon spirit (*Yang Măt Khei*) [30]. The hot-tempered *Yang Măt Nar* (Sun) is the deity of fertility that warms the earth and makes good quality rice. *Yang Măt Nar* also lights the way for people to go to the forest and fields. *Yang Măt Khei* (Moon) is fresh and always close to workers. Her regular movements become the rhythm of human life, crops and agricultural rituals. Therefore, according to Thủy, in Bahnar culture, “the sun and the moon often appear together, as one of the decorative motifs on paintings, *rong* houses, and patterns on wickerwork or textile products of the Ba Na people” [30]. Besides, the *yang of* Water (*yang Dak*) is beautiful with a sweet voice, friendly, loves to enjoy festivals, and is one of the favorite deities of the Bahnar people. In contrast, the God of Thunder (*Bok Glaih*) is imagined as a giant with a tall body and a fierce appearance, who might easily get angry and punish those who go against Bahnar traditional customs. The *yang* of Fire (*yang Unh*) is considered powerful because of his tremendous destructive ability.

The Bahnar interact not only with the surrounding physical environment, but also with spiritual agents and forces. For them, the natural spiritual powers can be both scary and familiar, forceful and negotiable. In describing the traditional beliefs of the Bahnar, Thủy [30] p. 53, from a Vietnamese perspective, proposes that ‘deep in their minds, the Ba Na people are terrified of supernatural forces, and they never ignore sacrificial rituals to find peace in life for their own community’. Going further, she generalizes the powerful existence of gods in the Bahnar mind. According to Thuy [30], p. 54, the Bahnar believe that:

the *yang* are the supreme beings who create all species, the universe, humans and determine the functions of everything…. Human beings, created by the *yang*, must be beholden to the gods and must not do anything that goes against the traditions of their ancestors. Because the gods assign humans to maintain the order that the gods have arranged, everything that is born from the gods will end in the gods. This is not anything complicated, but it is the ordinary expression of a living and general belief that the *yang* is everywhere and can see everything.

However, other Vietnamese ethnographers suggest that relations between the Bahnar and supernatural powers can be negotiable and interactive. According to Nguyễn and Nguyễn [37], the Bahnar exchanges with gods contain many interactive practices and efforts, including praying for support, promising (to hold an acknowledging ritual if the *yang* offer positive support), punishing (one’s enemies), fortune-telling (one’s positive or negative future), and gratefully acknowledging support from *yang*.

### 5.2. Bahnar exchanging and negotiating relations with *yang*

Nguyễn and Nguyễn [37] further conceptualize the relationship between the Bahnar and their traditional deities as reciprocal ‘exchanges’ (*cuộc giao tế -* in Vietnamese) between two parties. Indeed, in the ethnographers’ explanations, secular human beings always thirst for their own happiness but only have limited abilities to achieve it. Thus, they need to pray for the spirits to support humans’ aspirations (for wellbeing, survival, and flourishing crops). These mutual connections led to what Nguyễn and Nguyễn call special ‘exchanges’ between humans and *yang* spirits that are conducted via rituals, sacrifices, prayers, vows, or even via dreams. We observed, and our consultants in the Bahnar community confirmed to us, that all these methods are still actively practiced.

The Bahnar negotiate with *yang* via rituals and prayers. For example, if a person (X) gets ill because of losing his/her soul, which may have wandered away from their body, the family may hold a ritual by the edge of the forest to call out: ‘Oh X... don’t stay here anymore, don’t get lost anymore, follow us and go home, etc…’. If the patient’s family surmised that a *yang* had captured their relative’s soul, they could pray to and address *yang* directly in the ritual: ‘May the gods come down to drink wine and eat liver (of buffalo, horse, etc., depending on the promise). In the past, I (or my grandparents) promised to offer sacrifices to the gods but could not offer you in time, so here I am offering you; please stop asking or punishing me and give me good health’ [55], p. 107.

Sometimes Bahnar cultural experts are invited to travel to Hanoi, to the Vietnam National Village for Ethnic Culture and Tourism to perform a buffalo sacrifice ritual in heritage cultural performances there. When they do this, they are afraid that their homeland *yang* spirits may see this ritual and expect them to perform the same ritual back at the village. Since the buffalo sacrifice is done minimally once in a lifetime, or only in a critical situation, it is often postponed for a number of years. Therefore, the public performance of the ritual in Hanoi, in a folkloric setting, creates a tension that may affect the intended timing of the ritual back in the home village. This story further affirms the sacredness of the connections and exchanges the Bahnar people have with their *yang* and ancestral spirits, which may extend beyond the homeland to encompass urban settings.

This sacredness can be further explained in the concept of *tơl* in Bahnar beliefs. In fieldwork in 2015–16, the first author encountered the concept of *tơl* (v.) meaning to answer, or to promise the spirits a buffalo sacrifice. This term implies the relations and interactions of Bahnar with *yang* and/or ancestor spirits. When facing difficulties with health, exchange trading, or managing hill plantations, the Bahnar may perform a *tơl* ritual to ask for spiritual support. For example, every Bahnar family (couple), once in their lifetime, promises to offer their first-owned buffalo (or cow) for their ancestor spirits. The sacrifice can be fulfilled long afterwards and following particular omens. It could be the case that after some years, the couple found that their resources had increased from the first-owned buffalo; thus, they should offer the first one to their ancestors. Alternatively, if one gets ill, the fortune-tellers may say that he (or she) still owes his/her ancestor the first buffalo; the sacrifice then must be done to *ming yang* (v.) (to placate the spirits). In another case, to wish for a new successful crop, the Bahnar hold the *tơl* ritual to negotiate with spirits that, if the family receives the spirits’ support to have a good harvest, they will offer the sacrifice ceremony afterwards to *sơnoh* (v.) (to pay the spirits what has been promised).

Other instances of human-gods exchange relations are found in the agricultural rituals accompanying shifting cultivation. Traditionally, when cultivating on hill fields, the Bahnar conduct rituals at every step of a rice planting and harvesting (choosing land, clearing rice fields, burning trees on the chosen rice fields, planting, caring for the fields, and harvest). Condominas conceptualized the ritual system of the Bahnar (and other ethnic groups in the Central Highlands as “ritual technology” to inform the natural/agricultural spirits to ask for their agreement and support [56]. Accordingly, each ritual connects Bahnar hill farmers with their nature spirits (*yang dak* – spirit of water*; yang kong* – spirit of hills and mountains*; yang bri* – spirit of the forest*; yang phe* – spirit of rice) to pray for and negotiate a good harvest.

After clearing a new field of grass and trees, the Bahnar begin sowing rice. On the first day, the homeowner goes to the storehouse to get a portion of the rice seeds and brings them to the field. The husband prepares chicken and wine for the rice sowing ceremony *(et pôm choi)* and prays: ‘Today we sow rice, we ask the *yang* of thunder, the *yang* of pom, the *yang* of rice, the *yang* of mountains, and *the yang* of trees. You can eat chicken liver and drink wine. Please love us and give us lots of rice so we can play gongs and buy *xatok*’ (jars of rice wine) [48].

### 5.3. Animist rituals under Catholicism and Communism

The exposure of the Bahnar to French missionaries in 1848 inaugurated a period of significant change to Bahnar society caused by institutional forces [56]. In the Catholic Bahnar village of Kon K’tu, sacrificial rituals as described above were forbidden by the Church. In addition, government policies in the 20^th^ and 21^st^ century banned traditional shifting cultivation practices and corresponding customary laws which were central to the relationship between the Bahnar and their forests. The mass in-migration of lowlanders into the Central Highlands after 1950 further transformed the demographic composition of the space, limiting the Bahnar’s access to forest resources [4]. Sedentary farming methods were established by colonial, neo- and post-colonial regimes to abolish shifting cultivation, an assumed cause of deforestation in the Central Highlands. Since what the government and religious authorities saw as animist beliefs of the Bahnar shaped their daily interaction with the ecology and forest, their continuity was challenged. Moreover, the modern Communist authority has branded traditional animist practices of the Central Highlanders as superstitious and, sometimes, outlawed them [4]. In public schools and curriculum, Bahnar cultural practices and languages, like those of other ethnic minority groups in Vietnam, are only nominally included [57,58].

However, a recent ‘inculturation turn’ within the Catholic Church in Kon Tum Province recognizes local culture and attempts to integrate minority cultures into religious practices thus maintaining the agricultural ritual system and people’s connections with the gods. The ‘inculturation turn’ within global Catholicism initially started with the Second Vatican Council (1962-1965) that marked a dramatic turn in the Church’s view of the non-Catholic world and foreign cultures. In Kon Tum, evangelization and cultural preservation have proceeded hand in hand as a duty and a responsibility of the Church since Vatican II. The Catholic missionaries embraced the essence of minorities’ religious cosmologies as constituted by nature gods (*yang*) but introduced to them a new *Yang*: that is, Christ as the highest *Yang* – *Bă Yang* (God of Heaven) – above all other traditional *yang*. Instead of removing the indigenous agricultural ‘ritual technology’, the Church maintained this ritual system and rewrote the rites of crops into their Bahnar Catholic ritual practice book (*Hlabar Khop* – Bahnar songs of prayers). As discussed in Trần [59], p. 104.

*Hlabar Khop* also contains prayers for all the traditional planting activities: there are prayers for choosing land for cultivation (*rơih teh*); prayers to be said before cutting trees (*kăl long*), burning trees (*xoh muih*), or planting (*choi*); prayers for rain (*khop apinh dak mi*) and to stop rain (*khop apinh kơ prăng*); prayers to make a new rice store (*xum nao*) and at harvest (*kech ba*).

In this way, amidst governmental policies and Catholic ‘inculturation’, traditional Bahnar rituals centered on the agricultural cycle were not fully eradicated, but Christianized.

## 6. Mediums of human-*yang* interactions

As we observed in our field work, and as also reported in the literature, the Bahnar have multiple means of communication with *yang* and other spiritual entities, of which most prominent are artefacts such as the *gâng* (ritual pole), rituals including gong music (*tôh chĩng chênh*), and the *bơjâu* (ritual masters). The *gâng* pole constructed to anchor the buffalo sacfirice ceremony is the most important object in Bahnar culture, with many symbolic components (Fig 4). Traditionally, the Bahnar only erect and decorate the most complex and beautiful *gâng* when holding the buffalo sacrifice ritual, the highest offered animal, followed in descending order by goats, pigs, and chickens. Moreover, the Bahnar only offer buffalos on the most important occasions such as for the new building/restoration of the *rong* communal house, and for paying the spiritual debt of a man once in his lifetime. In the Bahnar communities that espouse Catholicism, such as Kon K’tu village where we conducted fieldwork, the Bahnar also build a *gâng* to offer to *Ba Yang* (Christ) every Christmas.

**Fig 3:**
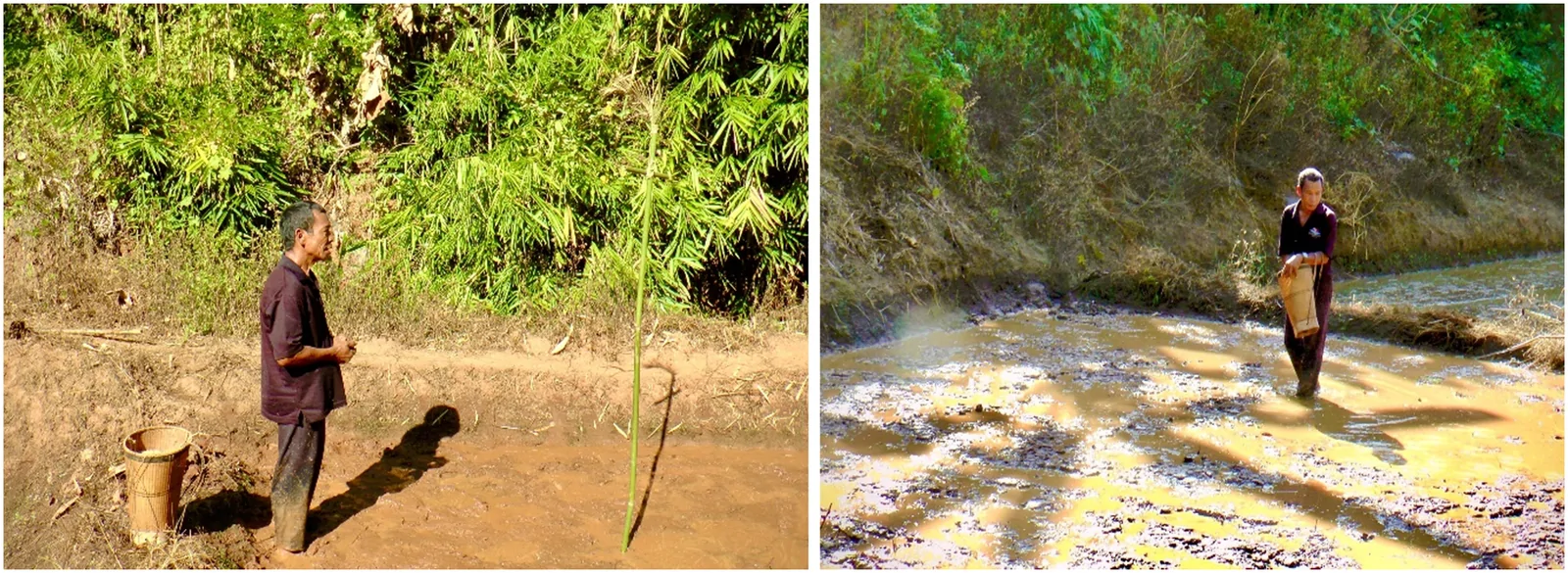
A Banh (L) praying for a new rice crop, and sowing rice grains (R) in the field after the ritual. Photo by the authors, published under the CC BY 4.0 license.

**Fig 4:**
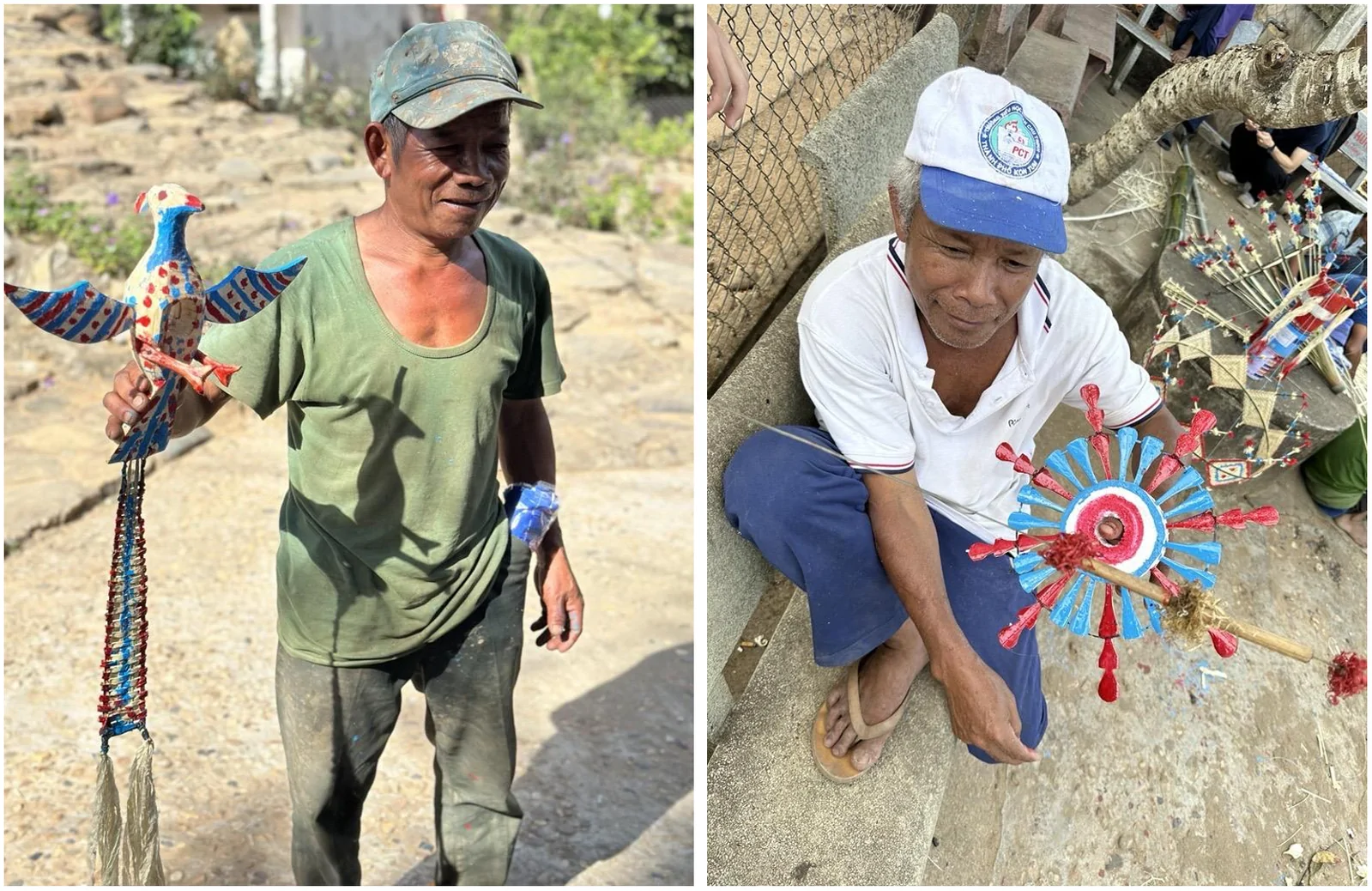
A Kal (L) with a carved wooden bird, and Chief A Ben with a sun symbol (R). Photos by the authors, published under the CC BY 4.0 license.

We observed and participated in the *gâng* ritual in 2022 and 2023 when dozens of men from Kon K’tu village constructed the sacred pole in about two days (Fig 2 and Fig 3). The pole we observed in December 2022 was commissioned by a local hotel owner, whose property borders the village and then installed in the central courtyard of the hotel. A bird carved from wood is attached to the upper part of the pole, representing a sacred symbol in Bahnar culture, which they call ‘earth eagle’ (*Kơ tơp ~ Kơ tru*), and which provides omens for forest activities. In December 2023, the village men constructed a pole in the village which they then transported and erected on the grounds of St. Mary’s Cathedral of Kon Tum city, in celebration of Christmas. We observed and participated in the preparation of the components, e.g., decorative bamboo fibers, wooden staves, woven bamboo objects that are suspended from the pole to represent (in a very abstract way) birds, and sun figurines (Fig 2 and Fig 4). Many pole components are painted, using a color palette of red, white, and light blue. The atmosphere during the *gâng* construction was festive, involving feasting, drinking, and singing, with nearly the entire village participating.

Besides the *gâng* ritual pole, in the rituals that the Bahnar perform to communicate with the gods or their ancestors’ spirits, gongs and gong music play crucial roles in sacred mediumship [59]. As Trần Cảnh Đào, the Vice-Director of the Department of Culture, Sports and Tourism of Lâm Đồng Province, put it: ‘Gongs are the sacred artifacts, used to transfer sacredness in the Highlanders’ ritual life’ [60], p. 80. The feeling of sacredness conveyed by gongs in their ritual context has been evocatively described by Nguyễn Thị Kim Vân, a researcher in the Department of Culture, Sports and Tourism of Gia Lai province. Gongs and gong music, he writes, are like the ‘incense-smoke in the ritual of the Vietnamese which helps people connect with gods and ancestors’ [61], p. 33

A Bahnar shaman or medium is called *bơjâu* (also *pơjâu,* the Jarai and Hrê people also use this term). According to Nguyễn and Nguyễn’s ethnography, there are four types of *po’jâu* categorized by particular spiritual skills of diagnosing and treating illness, namely: *po*’*jâu choitep* (the shaman who uses the lamp to find illness in a patient’s body), *po*’*jâu hơđa* (the shaman who uses his/her hand span to measure and find out the illness), *po*’*jâu plaih* (the shaman who uses his/her arm to measure and find out the illness), và *po*’*jâu po*’*tuh kơtap* (the shaman who uses a chicken egg to find out the illness) [37].

According to the field notes of the first author, shamans or ritual masters (*bơjâu*) play crucial roles in the traditional customs of the Bahnar, Jarai, and Brau ethnic communities in the Central Highlands of Vietnam. Being a *bơjâu*, in most cases, is not one’s primary intention but is caused by the influence of spiritual powers [62]. Thus, being a *bơjâu*, for many ritual masters, is fate, as ancestral spirits chose and offered them spiritual capacities to assume the responsibility of helping community members fix problems they may unexpectedly encounter. In the modern context, the *bơjâu* in Vietnam’s highlands experience complex situations that challenge their traditional roles. The state’s cultural policies of selective preservation consider shaman and shamanic practices as backward and superstitious. The Catholic church (as established among the Bahnar and Jarai) views traditional animistic rituals as *hơ mắt* (a pejorative Bahnar term meaning ‘satan(ic)’ or ‘devil(ish)’).

Thus, the highlands *bơjâu* currently playing their roles are faced with discussions about their expertise in the contradictory context of tradition, Catholicism, and modernity. Despite existing criticisms, the demand for services provided by *bơjâu* remains considerable among mountain communities. But modern critiques have effects on the ways shamans perform and interpret their activities. Many *bơjâu* in the Jarai and Bahnar communities talk about their skills (health care) as modern scientific ones (that do not only rely on holding rituals to pray to spirits). Privately, community members continue asking *bơjâu* to conduct spiritual shamanic practices, particularly in urgent cases. Once a woman in Kon K’tu, in her thirties, got ill for a long time and could not recover. She went to hospital and it turned out that she had cancer. In critical condition, she silently went to meet a *bơjâu* at a Bahnar traditional “*hơ mắt*” village, asking for spiritual help as the last hope to cure the disease. The spiritual treatment did not offer the magic as the woman died. Her “*hơ mắt*” act to ask for spiritual help received some criticism from the Catholic Kon K’tu villagers. However, some elders in the village sympathized with it as an understandable effort of the woman and her relatives in the traditional village to try all possible solutions. As Tran observes, there is continuing demand for animist curing rituals in the highlands communities, even as they adopt other religions such as Buddhism and Christianity [36]. Similarly, within Jarai villages like those in Ia Chim commune, notwithstanding the recent conversion of some young people to Catholicism, they continue to visit the village spiritual *bơjâu* for healing rituals when they or their children fall ill, as noted by the village chief.

To the Bahnar, animism is not only a way of knowing and conceptualizing the surrounding (spiritual) environment. The traditional system is also their way of thinking and behaving that follows animist moral principles. Animist understandings consequently affect people’s everyday behavior at home or in the forest. Animism as a moral worldview is recorded among other ethnic groups in Vietnam. Among the Katu, according to Århem [63], p. 114 ‘... people, spirits and the landscape are closely interconnected. The notion of spirits and the notion of landscape, or ‘nature’, are intriguingly interlinked; in many ways, the local concept of spirits corresponds to, or fuses with, the features of the landscape and its beings.’ The Katu view nature as imbued with agency and ‘mind’…Katu narratives reveal ‘a convergence in Katu thought between “morality” and “ecology”, where moral rules and ecological observances always walk hand in hand’ [63], pp. 114–115. In other words, as Århem puts it, the Katu ‘... seek to achieve in their dealings with the spirits is representative both of a specific cultural ethos, a moral attitude towards “nature”, and a profound practical understanding of the local forest environment’ [63], p. 135. We find this analysis of Katu practices to be very apt for cultural practices among the Bahnar.

**Fig 5:**
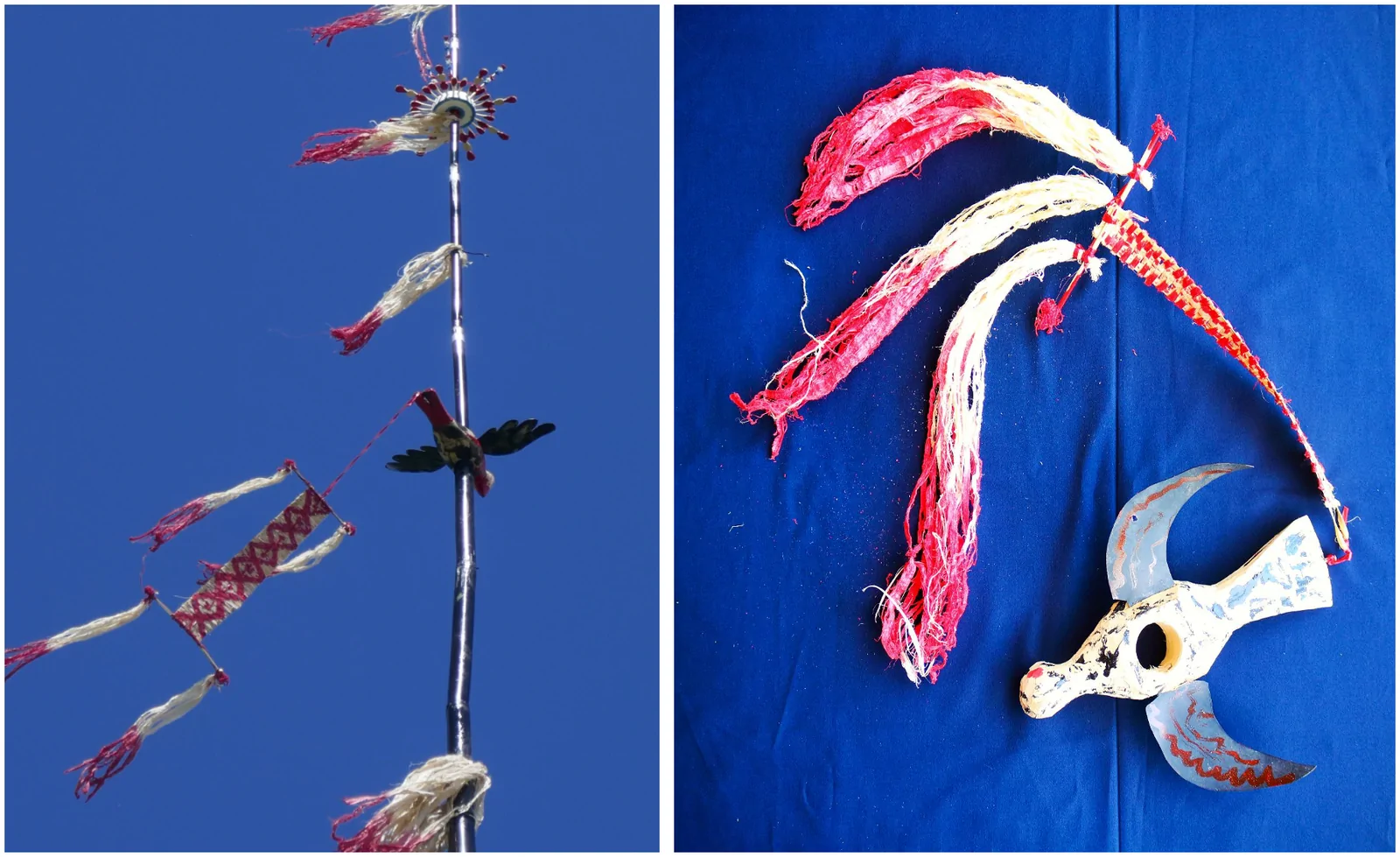
The symbolic wooden bird carving on the Bahnar *gâng* ceremonial pole. Photos by the authors, published under the CC BY 4.0 license.

The Bahnar’s perception of birds as omens provides insight into their moral and cultural attitudes of the agency of non-human beings. Indeed, in the Bahnar view, tangible forces of the spiritual world influence and guide human behaviors. This may be clearly observed in the case of birds’ calls and the potentialities of forest spirits. In Bahnar culture, there are auspicious birds (*xem lơng*) and birds that give bad omens (*xem kơni*) they might meet when traveling in the forest. Ellen [64], p. 575 describes similar bird omens among the Nuaulu people of Papua New Guinea: ‘...a bird appearing in a particular way at a particular juncture…may be the result of spirit intervention’. Like the Nuaulu, the Bahnar attend to birds’ vocalizations, as well as flight directions relative to people’s walking direction or their eye-gaze, as favorable or unfavorable portents for the journey. The Bahnar listen closely to the sounds of birds at specific locations (left side or right side of the path), which portends the potential success of the trip. Listening to the birds when traveling in the woods is crucial. They call the birds whose songs serve as omens *bơlang* (or *xem bơlang*). The act of consulting birds for omens is called *chơro bơlang* or *chơro xem* and is defined by Dourisboure as: “Consulter les oiseaux, aller écouter leur chant pour savoir ce qu’il annonce (supers.) [Consult the birds, listen to their song to find out what it announces (superstition)]” [44], p. 40, 61. If lucky, one would receive signals from favorable birds (*bơlang lơng*) and not from unfavorable or ‘sinister’ birds (*bơlang kơni*) to be confident about an upcoming trip [44], p. 40. Further displaying the symbolic power of birds, the Bahnar *gâng* ritual pole prominently displays a carving of a bird near the top, as the only animal figure included in the structure (Fig 5).

According to the Bahnar, invisible spirits and non-human agencies possess effective power, influencing human behavior in the forest. Like the Katu, the Bahnar see spiritual beings not as distant gods, but as connected with and ethically controlling daily activities. In the Bahnar-English dictionary [43], we find many terms denoting mythical beings or spirits, such as *gơhlŏu, gơhlâu* (‘an imaginary object that lives in trees and has [a] good smell’), *kơnŏk, yă kơnŏk* (‘name of a mythical woman who plays with present-day children’), *hui-hai* (‘name of imaginary people’), *hri, yang hri* (‘the spirit that makes rice grow’). Among those spirits, the Bahnar in Kon K’tu village have lively memories and engagement with the *hui-hai.* According to former chief A Ben and culture expert A Xom, *hui hai* (male) and *ya hui hai* (female) are believed to be real but invisible persons of miniature size. *Hui hai* hide in the forest and go down to the stream to catch crabs to eat when it rains. Even though the Bahnar know about *hui hai,* very few people see them, but typically see only their footprints. According to Bahnar tradition, the *hui hai* is petite but very strong and can carry large stones. It may throw stones at people who do or say something wrong in the forest. If people act appropriately, they need not worry. Thus, awareness of *hui hai* reminds people to behave carefully and appropriately while in the woods. This illustrates how sacred aspects of the forest guide the ways people interact with it. As Noth notes: ‘...mythological models of human ecology have been culturally transmitted in the form of narratives which instruct humans about their place in nature, telling them what they can, should, and must do with their natural environment’ [65], p. 334.

## 7. Conclusions

Animism as described in the ethnographic literature reveals a global distribution of unique local cosmologies and practices. Some of these traditional systems have been used as case studies to construct theories of animism, but many remain poorly documented, a gap we seek to fill with this report on the Bahnar ethnic minority in Vietnam’s Central Highlands. We describe Bahnar animism as an ongoing story that adapts to change and connects people to their land. Notwithstanding the adoption of Catholicism by many Bahnar, they still maintain and openly talk about their spiritual relationship with surrounding nature. The Bahnar have a relational view of the environment that invokes spiritual forces, especially those they call *yang*, and guides human conduct. Bahnar animism may thus be seen as a cultural pattern of thoughts and actions that helps to protect biodiversity, building upon centuries of successful and largely sustainable human interactions with the sacred forest environment [66]. Examples of environmental behaviors that are guided by Bahnar animistic understandings include forest wayfinding, river navigation, sustainable gathering of wild plants, carefully observing bird behaviors, seasonal rice planting and harvesting, swiddening, basket-making, vernacular architecture, hunting, ritual sacrifices, canoe building, rice wine production, and using plants as medicines.

The Bahnar animistic worldview is expressed across all Bahnar language genres, from legacy dictionaries written more than a century ago [44], to orally transmitted epic tales [67], traditional and contemporary popular songs [68], and modern digital media such as the Bahnar Talking Dictionary [69]. Ongoing efforts such as these attest to the priority that the Bahnar place on cultural revitalization [48,70]. Bahnar culture has also received some external recognition: in 2008, UNESCO recognized Vietnam’s ‘Space of Gong Culture’—which includes the Bahnar people along with other Central Highlands communities—in its Representative List of the Intangible Cultural Heritage of Humanity. Inclusion in this list affirms the essential value of the highlanders’ spiritual connections with surrounding mountainous landscapes and spirits, not merely as part of a nostalgic past but also as a practical method to transmit Bahnar environmental knowledge that enables them to continue caring for the forest today.

## Supporting information

S1 Appendix The Lexicon of Bahnar Animism. (Dourisboure, 1889; Guilleminet and Alberty, 1959–1963; Banker et al., 1972).
(DOCX)

S1 Checklist Tran et al. Inclusivity in global research questionnaire. (DOCX)

S1 File Human subjects checklist. (DOCX)
