# Supplementary material for: Yang, forest animism, and environmental perception among the Bahnar in the Central Highlands of Vietnam
Source: PLoS One. 2026 Aug 26;21(8):e0354453. doi: 10.1371/journal.pone.0354453 (PMC13513994; doi:10.1371/journal.pone.0354453)
Supplement: Supplementary file 1 [file pone.0354453.s001.docx]

| Appendix 1: The Lexicon of Bahnar Animism  (Dourisboure, 1889; Guilleminet and Alberty, 1959–1963; Banker et al, 1972) | | |
| --- | --- | --- |
| **Bahnar** | **Vietnamese translation** | **English translation/ interpretation** |
| Bodruh (n.) | N/A | Imaginary insect that causes dysentery and that the pythonisse (clairvoyant healer) makes a pretense of removing to cure the patient |
| Bok Glaih (n.) | Thần Sấm chớp | God of thunder |
| Bok Hoch (n.) | N/A | A giant mythical bird with a wide wingspan and a terrifying cry |
| Gơhlŏu / Gơhlâu (n.) | Thần trú ngụ trong cây | An imaginary object that lives in trees, has good smell |
| Gru / Guru (n.) | Linh hồn con người | Human-related spirits |
| Hodrĕ (n.) | Ác ma | An evil spirit |
| Hui Hai / Yă Hui Hai (n.) | N/A | Name of imaginary people, amazons |
| Kǐăk, Kǐek (n.) | Linh hồn người đã khuất | The souls of the dead |
| Kiĕk Wir (n.) | N/A | A tiger that is able to turn itself into a man |
| Kỏchít (v.) | Bị ám chết | To make perish, to perish |
| Kong Yang Long (n.) | Thần Cây | Spirit of Tree |
| Kơnŏk, Yă Kơnŏk (n.) | N/A | Name of a mythical woman who plays with present-day children |
| Mànes (n.) | Linh hồn tổ tiên | Ancestor spirits |
| Ming Yang (v.) | Tế | To placate the spirits, the term used when sacrifices are made |
| Mơsêh (v.) | Có phép thần thông | Have supernatural magic |
| Mơyang (v.) | Có năng lực siêu nhiên | Have supernatural powers |
| Pơjâu. (n.) | Thầy cúng / phù thuỷ | A shaman |
| Pơwir (v.) | N/A | To cause oneself to change into an animal or vice versa |
| Prao (n.) | Con rồng, quái thú | Dragon, monstrous serpent |
| Soi (v.) | Giao tiếp với linh hồn | To commune with the spirits, sacrifice |
| Sơkat (v.) | N/A | To speak with magical power, to speak and have something magical happen |
| Sơlỗ (n.) | N/A | A magic medicine that blinds the eyes of others for a while |
| Sơnoh (v.) | N/A | To pay to spirits what has been promised |
| Sơtôk (n.) | N/A | Type of wine jar which is the most expensive and has a spirit associated with it |
| Thŭ (n.) | Phép thần thông | Magical word used to make things happen |
| Tơl (v.) | N/A | To answer, to promise the spirits a sacrifice |
| Ư-ŏ-Pơgang Ư-ŏ (n.) | N/A | Type of medicine used to stop spirits from entering the village |
| Xỏnoh (n.) | Sự nguyền rủa | Debt allegedly contracted towards spirits by the violation or omission of a superstitious practice |
| Yang ~ Iāng (n.) | Linh hồn, vị thần | Spirit, deity, supernatural being |
| Yang Bri (n.) | Thần Rừng | Spirit of the Forest |
| Yang Dak (n.) | Thần Nước | Spirit of the Water |
| Yang Hri (n.) | Thần làm trổ lúa | Spirit that makes rice grow |
| Yang Kông (n.) | Thần Núi | Spirit of the Mountains |
| Yang Krong (n.) | Thần Sông | Spirit of the River |
| Yang Măt Khei (n.) | Thần Mặt trăng | Spirit of the Moon |
| Yang Măt Nar (n.) | Thần Mặt trời | Spirit of the Sun |
| Yang T'mo (n.) | Thần Đá | Spirit of the Rock |
| Yang Teh (n.) | Thần Đất | Spirit of the Earth |
| Yang Unh (n.) | Thần Lửa | Spirit of Fire |
| Yang Xơri (n.) | Thần Gạo | Spirit of Rice |
| Yang Zờn (n.) | Thần Thu hoạch | Spirit of the Harvest |
