# Supplementary material for: Yang, forest animism, and environmental perception among the Bahnar in the Central Highlands of Vietnam
Source: PLoS One. 2026 Aug 26;21(8):e0354453. doi: 10.1371/journal.pone.0354453 (PMC13513994; doi:10.1371/journal.pone.0354453)
Supplement: Supplementary file 4 [file pone.0354453.s004.docx]

Review Bahnar Animism

Reject

The article sets out to argue that animist representations of the Bahnar (Central Vietnam) serve to create moral relationships with the non-human environment and thus make Bahnar stewards of the nature they live in. However, the article, while showing that Bahnar everyday life resonates with non-human beings populating the forest, fails to provide evidence for this kind of stewardship. There is no description and analysis of practices that support sustainable use, avoid overuse or needless destruction of species and landscapes. There is also no detailed description how the practices and representations related to those invisible beings called yang regulate moral life among Bahnar – the authors simply mention good or bad behavior without any specification or sense for community morality.

1. Concepts

The article focuses a lot on words, and therefore, specific attention should be given to the words the authors use for their analysis.

One of the major problems is the tension between the aim to consider Bahnar cosmology/ontology in its own right and the proficient use of English terms that come with a problematic Eurocentric baggage which the authors do not reflect upon.

For instance, they translate the Bahnar concept yang mostly as spirit but sometimes as god. Both terms (especially their difference) are heavily influenced by Euro-American concepts that are in many ways in strong contrast to each other. The difficulty of translating yang is testimony to the confusion these translations provoke. However, the authors seem to ignore this problem.

Therefore, a much more detailed comment on how the term yang is understood by the Bahnar themselves is required. Are yang bodiless or bodied? Invisible or visible on specific occasions? Are they in this state always or sometimes? How do they relate to the material world – are they located or placeless, can they move or be moved? How do yang relate to invisible aspects of living human beings (“souls”) or to the dead? How do they make themselves known? The authors also mention ancestor spirits, but it is unclear what this means. Are they lineal or cognatic? Focused on the house or on several related households? Are there specialists addressing them? Which kind of beings (for instance, the hui hai) noted in the article are yang and which are not? Without even a glimpse of this knowledge, it is very difficult to know what the term means.

The elaboration on the apparent contradiction between the gods having absolute power over humans and the fact that they can be negotiated with is, however, much more interesting and to the point.

The authors also differentiate between “good” and “evil” gods. In most upland Southeast Asian societies, this metaphysical distinction is, to this reviewer’s understanding, inapplicable and misleading. Rather, “spirits” are more or less benevolent or dangerous. Maybe Bahnar are different, but the issue needs elaboration.

Also, the authors use “belief” when referring to Bahnar representations of spirits while they use “knowledge” when speaking about the Bahnar way of relating to the environment. This is a rather modernistic distinction between what is allegedly real (knowledge) and what only exists in people’s heads (belief). Yet the authors argue that animism is environmental knowledge – thus creating a contradiction in their use of concepts. The question is if this distinction corresponds to distinctions in Bahnar epistemology or if the authors project their own epistemological apparatus on the Bahnar. If the Bahnar do not make any such distinctions, it would be more appropriate to use more neutral terms such as “representations” or “assumptions”, or use “knowledge” throughout.

2. Method

The methods section is highly elaborate. However, while attempting to check all the boxes of responsible collaborative fieldwork, there is doubt if the authors take their methods and research epistemology seriously. For instance, they claim that they have considered their positionality, but reveal nothing about it. Positionality is important because it shapes the knowledge acquired – just mentioning it does not serve any purpose.

3. Theory

The authors claim that their material corroborates Ingold’s concept of animism. This is interesting, as Ingold is notoriously difficult to apply for analysis. Ingold, for instance, would abstain from notions such as “sacred” that imply a distinction between sacred and profane. Also, in the next section, the authors quote McNamara ea who explain that the person-like features of gods are “extensions we evolved to understand our human minds” – that is, they are imaginary projections of human experiences with their own kind. This is quite precisely the kind of interpretative/cognitive approach to the world that Ingold has been fighting against for decades. It is thus unclear how the authors solve the contradiction between theories that they have set up. In any case, both theoretical approaches are not elaborated upon or actually employed for analysis.

4. Data presentation

The authors claim that epics are “a medium of animist expression and environmental knowledge.” However, no evidence is given for this claim. There is a mere list of various unseen beings who are said to inhabit certain natural features, but there is no indication how this contains environmental knowledge. It is not even clear which function these epic tales have in Bahnar society. Are they considered historical truth or invention? Are they told in contexts that reinforce their alleged environmental or animist content?

Many of the ethnographic details that the authors give do not contain any plausible evidence for their main argument, that animism supports environmental knowledge and make Bahnar better stewards of the environment. The sacrifices described in 5.2, for instance, obviously negotiate well-being and health with spirits, but no connection with the environment is made. The authors mention agricultural rituals but once again this does neither imply environmental knowledge nor stewardship.

The same goes for the history of Bahnar animism under Catholicism and Communism. This would be inherently interesting, but again, the ethnography is too thin to allow for conclusions.

The authors describe ritual specialists in terms of “multispecies communities”. This is irritating, as the term multispecies is used for plants, animals, microbes or fungi, not for spirits. The references given also do not support the authors’ idioyncratic usage.

Rather late in the article, the authors start making an argument about the link between morality and ecology, quoting Nikolas Arhem. While Arhem actually gives plenty of detail in order to argue his point, the authors choose bird omens as an example. However, bird omens – which are common in the region – do not have a moral character, but are simply premonitory. They neither punish bad behavior, nor do they in any way serve to protect the environment.

Another example concerns small, mostly invisible people who throw stones at people who do not behave properly in the forest. There is, however, no explanation what “good” or “bad” behavior consists of and how it relates to the ecology.

Both in the abstract and on p. 34 there is a long list of activities allegedly guided by “Bahnar animistic beliefs”, but none of this is explained.

In future attempts, the authors should focus on a few examples of ecologically relevant behavior and the animist assumptions shaping them, describe and analyse them in depth, instead of filling their text with a plenitude of superficial and irrelevant topics.
