# Supplementary material for: Yang, forest animism, and environmental perception among the Bahnar in the Central Highlands of Vietnam
Source: PLoS One. 2026 Aug 26;21(8):e0354453. doi: 10.1371/journal.pone.0354453 (PMC13513994; doi:10.1371/journal.pone.0354453)
Supplement: Peer review history

# Peer review: Decision Letter 0

**DOI:** 10.1371/journal.pone.0354453.r001

**Authors:** Abebayehu Aticho

29 Aug 2025

PONE-D-25-33740

Animism as environmental knowledge in Vietnam: Bahnar spiritual-ecological beliefs

PLOS ONE

Dear Prof.Harrison,

Thank you for submitting your manuscript to PLOS ONE. After careful consideration, we feel that it has merit but does not fully meet PLOS ONE’s publication criteria as it currently stands. Therefore, we invite you to submit a revised version of the manuscript that addresses the points raised during the review process.

The authors must address the following core issues to make the manuscript viable:

1. Reframe or Substantiate the Core Argument: The authors face a choice:
  - Option A (Narrowing the Focus): Drastically narrow the paper's claim. Instead of arguing for environmental stewardship, reframe it as an exploration of Bahnar animist cosmology and its relationship with the *perceived*  environment. This would align better with the current descriptive data.
  - Option B (Substantiating the Claim): Provide the missing robust evidence for stewardship. This would require new analysis and writing focused on specific, detailed examples of practices that directly prevent overuse, manage resources, or punish ecological transgressions, explicitly linking them to animist principles. This is the more difficult but potentially more impactful path.
2. Deepen Ethnographic and Conceptual Analysis: The paper must move beyond listing beliefs and practices. It requires:
  - A thorough, nuanced exploration of the key term *yang* , addressing all the questions raised by Reviewer 2 (bodily form, location, relation to the dead, etc.).
  - A critical examination of the terms "spirit," "god," "belief," and "knowledge," explaining their use and potential limitations in translation.
  - A clear explanation of Bahnar morality: what constitutes "good" and "bad" behavior in this context, specifically in relation to the non-human world.
3. Strengthen Theoretical Cohesion: The authors must resolve the theoretical contradiction pointed out by Reviewer 2. They cannot claim to follow Ingold's relational ontology while also citing cognitive theories that contradict it. They must choose a theoretical lens and apply it consistently throughout the analysis or clearly explain how they are synthesizing these perspectives.
4. Address Specific Suggestions from Both Reviewers:
  - Expand the historical context slightly (R1).
  - Consider alternative terminology to "animism" or justify its use more robustly (R1).
  - Add a brief comparative note on Southeast Asian parallels (R1).
  - Elaborate on positionality—how the authors' own backgrounds shaped the research (R2).

This manuscript has potential but in its current form, it does not fulfill its ambitious paper. The decision is Major Revision, with the hope that the authors are able to address all comments of reviewer 2 (rejection) points by point. The revisions required are not minor; they are fundamental and concern the paper's core argument, methodological depth, and theoretical coherence. The author's response must convincingly outline how they will address these significant concerns. A resubmitted manuscript will need to be re-evaluated, likely by both original reviewers if possible, to ensure the substantial issues have been resolved.

Please submit your revised manuscript by Oct 13 2025 11:59PM5. If you will need more time than this to complete your revisions, please reply to this message or contact the journal office at plosone@plos.org. When you're ready to submit your revision, log on to https://www.editorialmanager.com/pone/ and select the 'Submissions Needing Revision' folder to locate your manuscript file.

Please include the following items when submitting your revised manuscript:

- A rebuttal letter that responds to each point raised by the academic editor and reviewer(s). You should upload this letter as a separate file labeled 'Response to Reviewers'.
- A marked-up copy of your manuscript that highlights changes made to the original version. You should upload this as a separate file labeled 'Revised Manuscript with Track Changes'.
- An unmarked version of your revised paper without tracked changes. You should upload this as a separate file labeled 'Manuscript'.

If you would like to make changes to your financial disclosure, please include your updated statement in your cover letter. Guidelines for resubmitting your figure files are available below the reviewer comments at the end of this letter.

If applicable, we recommend that you deposit your laboratory protocols in protocols.io to enhance the reproducibility of your results. Protocols.io assigns your protocol its own identifier (DOI) so that it can be cited independently in the future. For instructions see: https://journals.plos.org/plosone/s/submission-guidelines#loc-laboratory-protocols. Additionally, PLOS ONE offers an option for publishing peer-reviewed Lab Protocol articles, which describe protocols hosted on protocols.io. Read more information on sharing protocols at https://plos.org/protocols?utm_medium=editorial-email&utm_source=authorletters&utm_campaign=protocols.

We look forward to receiving your revised manuscript.

Kind regards,

Abebayehu Aticho (PhD, Associate Professor)

Academic Editor

PLOS ONE

Journal Requirements:

When submitting your revision, we need you to address these additional requirements.

1. Please ensure that your manuscript meets PLOS ONE's style requirements, including those for file naming. The PLOS ONE style templates can be found at

https://journals.plos.org/plosone/s/file?id=wjVg/PLOSOne_formatting_sample_main_body.pdf and

https://journals.plos.org/plosone/s/file?id=ba62/PLOSOne_formatting_sample_title_authors_affiliations.pdf.

2. Please include a complete copy of PLOS’ questionnaire on inclusivity in global research in your revised manuscript. Our policy for research in this area aims to improve transparency in the reporting of research performed outside of researchers’ own country or community. The policy applies to researchers who have travelled to a different country to conduct research, research with Indigenous populations or their lands, and research on cultural artefacts. The questionnaire can also be requested at the journal’s discretion for any other submissions, even if these conditions are not met.  Please find more information on the policy and a link to download a blank copy of the questionnaire here: https://journals.plos.org/plosone/s/best-practices-in-research-reporting. Please upload a completed version of your questionnaire as Supporting Information when you resubmit your manuscript.”

3. In your Methods section, please provide additional information regarding the permits you obtained for the work. Please ensure you have included the full name of the authority that approved the field site access and, if no permits were required, a brief statement explaining why.

4. Thank you for stating the following financial disclosure:

‘VinUniversity seed grant, valued at $20K USD over two years.”.

Please state what role the funders took in the study.  If the funders had no role, please state: "The funders had no role in study design, data collection and analysis, decision to publish, or preparation of the manuscript."

If this statement is not correct you must amend it as needed.

Please include this amended Role of Funder statement in your cover letter; we will change the online submission form on your behalf.

5. Thank you for stating the following in the Acknowledgments Section of your manuscript:

“The research was supported by a research grant from the Center for Environmental Intelligence (VinUniversity), and an expedition grant from The Explorers Club, which we gratefully acknowledge. The funders had no role in study design, data collection and analysis, decision to publish, or preparation of the manuscript.”

We note that you have provided additional information within the Acknowledgements Section that is not currently declared in your Funding Statement. Please note that funding information should not appear in the Acknowledgments section or other areas of your manuscript. We will only publish funding information present in the Funding Statement section of the online submission form.

Please remove any funding-related text from the manuscript and let us know how you would like to update your Funding Statement. Currently, your Funding Statement reads as follows:

“VinUniversity seed grant, valued at $20K USD over two years.”

Please include your amended statements within your cover letter; we will change the online submission form on your behalf.

6. Please include captions for your Supporting Information files at the end of your manuscript, and update any in-text citations to match accordingly. Please see our Supporting Information guidelines for more information: http://journals.plos.org/plosone/s/supporting-information.

7. We note that Figures 1 in your submission contain [map/satellite] images which may be copyrighted. All PLOS content is published under the Creative Commons Attribution License (CC BY 4.0), which means that the manuscript, images, and Supporting Information files will be freely available online, and any third party is permitted to access, download, copy, distribute, and use these materials in any way, even commercially, with proper attribution. For these reasons, we cannot publish previously copyrighted maps or satellite images created using proprietary data, such as Google software (Google Maps, Street View, and Earth). For more information, see our copyright guidelines: http://journals.plos.org/plosone/s/licenses-and-copyright.

u to either (1) present written permission from the copyright holder to publish these figures specifically under the CC BY 4.0 license, or (2) remove the figures from your submission:

a. You may seek permission from the original copyright holder of Figures 1 to publish the content specifically under the CC BY 4.0 license.

We recommend that you contact the original copyright holder with the Content Permission Form (http://journals.plos.org/plosone/s/file?id=7c09/content-permission-form.pdf) and the following text:

“I request permission for the open-access journal PLOS ONE to publish XXX under the Creative Commons Attribution License (CCAL) CC BY 4.0 (http://creativecommons.org/licenses/by/4.0/). Please be aware that this license allows unrestricted use and distribution, even commercially, by third parties. Please reply and provide explicit written permission to publish XXX under a CC BY license and complete the attached form.”

Please upload the completed Content Permission Form or other proof of granted permissions as an "Other" file with your submission.

In the figure caption of the copyrighted figure, please include the following text: “Reprinted from [ref] under a CC BY license, with permission from [name of publisher], original copyright [original copyright year].”

b. If you are unable to obtain permission from the original copyright holder to publish these figures under the CC BY 4.0 license or if the copyright holder’s requirements are incompatible with the CC BY 4.0 license, please either i) remove the figure or ii) supply a replacement figure that complies with the CC BY 4.0 license. Please check copyright information on all replacement figures and update the figure caption with source information. If applicable, please specify in the figure caption text when a figure is similar but not identical to the original image and is therefore for illustrative purposes only.

The following resources for replacing copyrighted map figures may be helpful:

USGS National Map Viewer (public domain): http://viewer.nationalmap.gov/viewer/

The Gateway to Astronaut Photography of Earth (public domain): http://eol.jsc.nasa.gov/sseop/clickmap/

Maps at the CIA (public domain): https://www.cia.gov/library/publications/the-world-factbook/index.html and https://www.cia.gov/library/publications/cia-maps-publications/index.html

NASA Earth Observatory (public domain): http://earthobservatory.nasa.gov/

Landsat: http://landsat.visibleearth.nasa.gov/

USGS EROS (Earth Resources Observatory and Science (EROS) Center) (public domain): http://eros.usgs.gov/#

Natural Earth (public domain): http://www.naturalearthdata.com/

8. We note that Figure 3& 4 includes an image of a [patient / participant / in the study].

As per the PLOS ONE policy (http://journals.plos.org/plosone/s/submission-guidelines#loc-human-subjects-research) on papers that include identifying, or potentially identifying, information, the individual(s) or parent(s)/guardian(s) must be informed of the terms of the PLOS open-access (CC-BY) license and provide specific permission for publication of these details under the terms of this license. Please download the Consent Form for Publication in a PLOS Journal (http://journals.plos.org/plosone/s/file?id=8ce6/plos-consent-form-english.pdf). The signed consent form should not be submitted with the manuscript, but should be securely filed in the individual's case notes. Please amend the methods section and ethics statement of the manuscript to explicitly state that the patient/participant has provided consent for publication: “The individual in this manuscript has given written informed consent (as outlined in PLOS consent form) to publish these case details”.

If you are unable to obtain consent from the subject of the photograph, you will need to remove the figure and any other textual identifying information or case descriptions for this individual."

9. If the reviewer comments include a recommendation to cite specific previously published works, please review and evaluate these publications to determine whether they are relevant and should be cited. There is no requirement to cite these works unless the editor has indicated otherwise.

Additional Editor Comments (if provided):

The authors must address the following core issues to make the manuscript viable:

1. Reframe or Substantiate the Core Argument: The authors face a choice:

o Option A (Narrowing the Focus): Drastically narrow the paper's claim. Instead of arguing for environmental stewardship, reframe it as an exploration of Bahnar animist cosmology and its relationship with the perceived environment. This would align better with the current descriptive data.

o Option B (Substantiating the Claim): Provide the missing robust evidence for stewardship. This would require new analysis and writing focused on specific, detailed examples of practices that directly prevent overuse, manage resources, or punish ecological transgressions, explicitly linking them to animist principles. This is the more difficult but potentially more impactful path.

2. Deepen Ethnographic and Conceptual Analysis: The paper must move beyond listing beliefs and practices. It requires:

o A thorough, nuanced exploration of the key term yang, addressing all the questions raised by Reviewer 2 (bodily form, location, relation to the dead, etc.).

o A critical examination of the terms "spirit," "god," "belief," and "knowledge," explaining their use and potential limitations in translation.

o A clear explanation of Bahnar morality: what constitutes "good" and "bad" behavior in this context, specifically in relation to the non-human world.

3. Strengthen Theoretical Cohesion: The authors must resolve the theoretical contradiction pointed out by Reviewer 2. They cannot claim to follow Ingold's relational ontology while also citing cognitive theories that contradict it. They must choose a theoretical lens and apply it consistently throughout the analysis or clearly explain how they are synthesizing these perspectives.

4. Address Specific Suggestions from Both Reviewers:

o Expand the historical context slightly (R1).

o Consider alternative terminology to "animism" or justify its use more robustly (R1).

o Add a brief comparative note on Southeast Asian parallels (R1).

o Elaborate on positionality—how the authors' own backgrounds shaped the research (R2).

Conclusion:

This manuscript has potential but in its current form, it does not fulfill its ambitious paper. The decision is Major Revision, with the hope that the authors are able to address all comments of reviewer 2 (rejection) points by point. The revisions required are not minor; they are fundamental and concern the paper's core argument, methodological depth, and theoretical coherence. The author's response must convincingly outline how they will address these significant concerns. A resubmitted manuscript will need to be re-evaluated, likely by both original reviewers if possible, to ensure the substantial issues have been resolved.

[Note: HTML markup is below. Please do not edit.]

Reviewers' comments:

Reviewer's Responses to Questions

**Comments to the Author**

1. Is the manuscript technically sound, and do the data support the conclusions?

The manuscript must describe a technically sound piece of scientific research with data that supports the conclusions. Experiments must have been conducted rigorously, with appropriate controls, replication, and sample sizes. The conclusions must be drawn appropriately based on the data presented.

Reviewer #1: Yes

Reviewer #2: No

**********

2. Has the statistical analysis been performed appropriately and rigorously?

Reviewer #1: Yes

Reviewer #2: N/A

**********

3. Have the authors made all data underlying the findings in their manuscript fully available?

The PLOS Data policy requires authors to make all data underlying the findings described in their manuscript fully available without restriction, with rare exception (please refer to the Data Availability Statement in the manuscript PDF file). The data should be provided as part of the manuscript or its supporting information, or deposited to a public repository. For example, in addition to summary statistics, the data points behind means, medians and variance measures should be available. If there are restrictions on publicly sharing data—e.g. participant privacy or use of data from a third party—those must be specified.

Reviewer #1: Yes

Reviewer #2: No

**********

4. Is the manuscript presented in an intelligible fashion and written in standard English?

PLOS ONE does not copyedit accepted manuscripts, so the language in submitted articles must be clear, correct, and unambiguous. Any typographical or grammatical errors should be corrected at revision, so please note any specific errors here.

Reviewer #1: Yes

Reviewer #2: Yes

**********

5. Review Comments to the Author

Please use the space provided to explain your answers to the questions above. You may also include additional comments for the author, including concerns about dual publication, research ethics, or publication ethics. (Please upload your review as an attachment if it exceeds 20,000 characters)

Reviewer #1: This is a solid article that is well written, well organized, and generally well-argued. It does appear that the authors have approached their subject with a considerable degree of sensitivity and appropriate cautions with respect to dealing with the population whose spiritual beliefs and practices they are examining. They have appropriately laid out their approach, their practices, and the mechanisms by which they engaged with local collaborators. Thus, it seems that they have taken all of the correct steps in conducting their research.

They do a solid job of giving an overview of Bahnar practices and beliefs and of situating them within the rather complicated historical, religious, and political landscape within which the Bahnar live and operate. Indeed, the pressures of missionary activity, of the modern Catholic Church, and of the contemporary Vietnamese communist state have all impacted the community and its beliefs in various ways and the article makes note of this, which is nice to see.

While the discussion of “animism” is solid, I would suggest that it is a bit limiting. There is brief acknowledgement that the terms is sometimes viewed as problematic and not used by some scholars, and I think given this, that the authors might have explored alternative terminology. Since “animism” carries a rather significant amount of cultural baggage - related to the kinds of dismissive attitudes of the Vietnamese state regarding ethnic minority populations - the authors might have considered perhaps using terms like “folk religion” or “traditional spiritual beliefs” or something along those lines. I do understand that “animism” captures more specifically the belief regarding the ways in which non-human and even material objects can have animating elements, but I still think the term is a bit loaded.

Secondly, while there are one or two very brief comparative observations, I guess I’d like to see at least brief attention to situating the Bahnar beliefs within a larger Southeast Asian context (or even just mainland Southeast Asian context). This would reveal the degree to which the Bahnar beliefs, rituals, and practices parallel those found across many ethnic groups and populations across both mainland and island Southeast Asia. Such a comparative comment (at least) would make clear that the Bahnar beliefs/practices are incredibly widespread in the region, even if they are quite different from those practiced by lowland Vietnamese. It would, I think, somehow “mainstream” Bahnar practices as less of an outlier, and more of a very common practice when seen across the broader region.

Third, the historical section is quite brief, and written in a rather passive style. This is not a big deal and I get that this article is not a history of the Bahnar, but this might be a bit more robust (perhaps another paragraph). Such an expanded paragraph could acknowledge the true impacts of the American war, which were pretty substantial for populations in the Central Highlands, as well as the integrative practices of the contemporary Vietnamese state. The latter are given some attention, though perhaps a bit too lightly.

Finally, while the abstract and the conclusion make mention of how a study of Bahnar practices and beliefs might lead them to ecological preservation and conservation practices, the article barely touches on this, much less actually explores it. In short, while it is possible that their beliefs might translate into preservationist practices, this theory is not explored nor really tested. This is a bit disappointing, because, of course, this is one of the standard claims about groups that are seen as being closer to nature or more in tune with the environment. I’d suggest that the authors make this section of the paper more robust if they are going to make those claims.

Overall, though this is a solid paper on an important ethnic community in Vietnam, and it is presented in an accessible manner that even non-specialists would enjoy and benefit from.

Reviewer #2: The article sets out to argue that animist representations of the Bahnar (Central Vietnam) serve to create moral relationships with the non-human environment and thus make Bahnar stewards of the nature they live in. However, the article, while showing that Bahnar everyday life resonates with non-human beings populating the forest, fails to provide evidence for this kind of stewardship. There is no description and analysis of practices that support sustainable use, avoid overuse or needless destruction of species and landscapes. There is also no detailed description how the practices and representations related to those invisible beings called yang regulate moral life among Bahnar – the authors simply mention good or bad behavior without any specification or sense for community morality.

1. Concepts

The article focuses a lot on words, and therefore, specific attention should be given to the words the authors use for their analysis.

One of the major problems is the tension between the aim to consider Bahnar cosmology/ontology in its own right and the proficient use of English terms that come with a problematic Eurocentric baggage which the authors do not reflect upon.

For instance, they translate the Bahnar concept yang mostly as spirit but sometimes as god. Both terms (especially their difference) are heavily influenced by Euro-American concepts that are in many ways in strong contrast to each other. The difficulty of translating yang is testimony to the confusion these translations provoke. However, the authors seem to ignore this problem.

Therefore, a much more detailed comment on how the term yang is understood by the Bahnar themselves is required. Are yang bodiless or bodied? Invisible or visible on specific occasions? Are they in this state always or sometimes? How do they relate to the material world – are they located or placeless, can they move or be moved? How do yang relate to invisible aspects of living human beings (“souls”) or to the dead? How do they make themselves known? The authors also mention ancestor spirits, but it is unclear what this means. Are they lineal or cognatic? Focused on the house or on several related households? Are there specialists addressing them? Which kind of beings (for instance, the hui hai) noted in the article are yang and which are not? Without even a glimpse of this knowledge, it is very difficult to know what the term means.

The elaboration on the apparent contradiction between the gods having absolute power over humans and the fact that they can be negotiated with is, however, much more interesting and to the point.

The authors also differentiate between “good” and “evil” gods. In most upland Southeast Asian societies, this metaphysical distinction is, to this reviewer’s understanding, inapplicable and misleading. Rather, “spirits” are more or less benevolent or dangerous. Maybe Bahnar are different, but the issue needs elaboration.

Also, the authors use “belief” when referring to Bahnar representations of spirits while they use “knowledge” when speaking about the Bahnar way of relating to the environment. This is a rather modernistic distinction between what is allegedly real (knowledge) and what only exists in people’s heads (belief). Yet the authors argue that animism is environmental knowledge – thus creating a contradiction in their use of concepts. The question is if this distinction corresponds to distinctions in Bahnar epistemology or if the authors project their own epistemological apparatus on the Bahnar. If the Bahnar do not make any such distinctions, it would be more appropriate to use more neutral terms such as “representations” or “assumptions”, or use “knowledge” throughout.

2. Method

The methods section is highly elaborate. However, while attempting to check all the boxes of responsible collaborative fieldwork, there is doubt if the authors take their methods and research epistemology seriously. For instance, they claim that they have considered their positionality, but reveal nothing about it. Positionality is important because it shapes the knowledge acquired – just mentioning it does not serve any purpose.

3. Theory

The authors claim that their material corroborates Ingold’s concept of animism. This is interesting, as Ingold is notoriously difficult to apply for analysis. Ingold, for instance, would abstain from notions such as “sacred” that imply a distinction between sacred and profane. Also, in the next section, the authors quote McNamara ea who explain that the person-like features of gods are “extensions we evolved to understand our human minds” – that is, they are imaginary projections of human experiences with their own kind. This is quite precisely the kind of interpretative/cognitive approach to the world that Ingold has been fighting against for decades. It is thus unclear how the authors solve the contradiction between theories that they have set up. In any case, both theoretical approaches are not elaborated upon or actually employed for analysis.

4. Data presentation

The authors claim that epics are “a medium of animist expression and environmental knowledge.” However, no evidence is given for this claim. There is a mere list of various unseen beings who are said to inhabit certain natural features, but there is no indication how this contains environmental knowledge. It is not even clear which function these epic tales have in Bahnar society. Are they considered historical truth or invention? Are they told in contexts that reinforce their alleged environmental or animist content?

Many of the ethnographic details that the authors give do not contain any plausible evidence for their main argument, that animism supports environmental knowledge and make Bahnar better stewards of the environment. The sacrifices described in 5.2, for instance, obviously negotiate well-being and health with spirits, but no connection with the environment is made. The authors mention agricultural rituals but once again this does neither imply environmental knowledge nor stewardship.

The same goes for the history of Bahnar animism under Catholicism and Communism. This would be inherently interesting, but again, the ethnography is too thin to allow for conclusions.

The authors describe ritual specialists in terms of “multispecies communities”. This is irritating, as the term multispecies is used for plants, animals, microbes or fungi, not for spirits. The references given also do not support the authors’ idioyncratic usage.

Rather late in the article, the authors start making an argument about the link between morality and ecology, quoting Nikolas Arhem. While Arhem actually gives plenty of detail in order to argue his point, the authors choose bird omens as an example. However, bird omens – which are common in the region – do not have a moral character, but are simply premonitory. They neither punish bad behavior, nor do they in any way serve to protect the environment.

Another example concerns small, mostly invisible people who throw stones at people who do not behave properly in the forest. There is, however, no explanation what “good” or “bad” behavior consists of and how it relates to the ecology.

Both in the abstract and on p. 34 there is a long list of activities allegedly guided by “Bahnar animistic beliefs”, but none of this is explained.

In future attempts, the authors should focus on a few examples of ecologically relevant behavior and the animist assumptions shaping them, describe and analyse them in depth, instead of filling their text with a plenitude of superficial and irrelevant topics.

**********

6. PLOS authors have the option to publish the peer review history of their article (what does this mean?). If published, this will include your full peer review and any attached files.

If you choose “no”, your identity will remain anonymous but your review may still be made public.

**Do you want your identity to be public for this peer review?**  For information about this choice, including consent withdrawal, please see our Privacy Policy.

Reviewer #1: **Yes:** George Dutton

Reviewer #2: No

**********

[NOTE: If reviewer comments were submitted as an attachment file, they will be attached to this email and accessible via the submission site. Please log into your account, locate the manuscript record, and check for the action link "View Attachments". If this link does not appear, there are no attachment files.]

While revising your submission, please upload your figure files to the Preflight Analysis and Conversion Engine (PACE) digital diagnostic tool, https://pacev2.apexcovantage.com/. PACE helps ensure that figures meet PLOS requirements. To use PACE, you must first register as a user. Registration is free. Then, login and navigate to the UPLOAD tab, where you will find detailed instructions on how to use the tool. If you encounter any issues or have any questions when using PACE, please email PLOS at figures@plos.org. Please note that Supporting Information files do not need this step.

Attachment Submitted filename: Review Bahnar Animism.docx

# Author comment: Author response to Decision Letter 1

**DOI:** 10.1371/journal.pone.0354453.r002

2 Mar 2026

Dear PLOS ONE editor,

We thank you and the two peer reviewers for a careful reading of our manuscript and for constructive comments. We have thoroughly revised the manuscript in response to each point raised. All changes are indicated in the tracked-changes version we uploaded. Below we respond (**our responses are starred**) point-by-point to each comment from you, as editor, and then from each reviewer.

A. Editor’s summary comments:

The authors must address the following core issues to make the manuscript viable:

1. Reframe or Substantiate the Core Argument: The authors face a choice:

o Option A (Narrowing the Focus): Drastically narrow the paper's claim. Instead of arguing for environmental stewardship, reframe it as an exploration of Bahnar animist cosmology and its relationship with the perceived environment. This would align better with the current descriptive data.

o Option B (Substantiating the Claim): Provide the missing robust evidence for stewardship. This would require new analysis and writing focused on specific, detailed examples of practices that directly prevent overuse, manage resources, or punish ecological transgressions, explicitly linking them to animist principles. This is the more difficult but potentially more impactful path.

**We have reframed the argument as per Option A, by considerably narrowing the focus and adding more supporting empirical and theoretical evidence. The paper’s focus is now on Bahnar animist cosmology as a body of knowledge that guides the Bahnar people’s relationship with the environment. We briefly suggest that this may connect to environmental stewardship, and also contribute to the literature on that topic, but we do not frame this as a claim or hypothesis. We do observe: “Furthermore, we find that Bahnar animism creates a mindset that encourages the community to protect and steward its natural environment” (lines 584-585)

**We also revised the title, so that the focus is clearly on the Bahnar concept of “yang”, and environmental perception/knowledge. Our title and claim now align well with the empirical data we present.

2. Deepen Ethnographic and Conceptual Analysis: The paper must move beyond listing beliefs and practices. It requires:

o A thorough, nuanced exploration of the key term yang, addressing all the questions raised by Reviewer 2 (bodily form, location, relation to the dead, etc.).

**We added new material that thoroughly exlplicates the term “yang”, and also contextualizes it by showing how it has been defined/glossed in the history of lexicography and ethnography by leading authors (Guilleminet, Dourisboure, Banker et al, Dournes). As some of these sources were written in French, we use selected direct quotes in French (followed by English translations) to illustrate how French authors have attempted to translate, gloss and define the term “yang”, which lacks a direct synonym/equivalent in French and other languages, and is thus established as an important emic concept in Bahnar. We specifically show how the form, gender, location, and relation to the dead and living have been described by ethnographers/lexicographers, and also how they are described and narrated by the Bahnar people today.

o A critical examination of the terms "spirit," "god," "belief," and "knowledge," explaining their use and potential limitations in translation.

Re: “knowledge” vs. “belief” we follow Ingold (2000) in using “knowledge” as the operative term to denote perceptual engagement with the environment, including yang spirits. We only use “belief” when referring to legacy ethnographies or religious perspectives that frame Bahnar cosmology as subjective (line 710).

We include this statement: (lines 217-219) “Contemporary anthropology generally avoids “belief” because it is an external analytic category implying subjective conviction, and prefers, as we do, “knowledge,” understood as experiential and relational engagement with the world. In Bahnar society, spirits are not objects of belief but components of lived environmental knowledge.”

**Re: "spirit," "god"; we consistently use “spirit” as the best translation of yang, while motivating why that is the case, based on prior ethnographic literature in French, English, and Vietnamese. We use “god” only when referring to/paraphrasing some legacy ethnographic texts, or for deities that are given proper names. We also sometimes use “deities”, in a generic sense, and with explanation. We strive for consistency across all uses, with the emic term yang as our most common and consistent usage, now also included in the paper’s title.

o A clear explanation of Bahnar morality: what constitutes "good" and "bad" behavior in this context, specifically in relation to the non-human world.

**We have added specific examples of proscribed or prescribed Bahnar behaviors, especially in terms of actions in the forest landscape (lines 154-170, 818-823, 833-837, 887-891) for example, not talking loudly in the forest, not taking more than is needed, attending to bird omens and reacting accordingly, making appropriate animal sacrifices, performing the proper rituals prior to stages of planting or harvest.

3. Strengthen Theoretical Cohesion: The authors must resolve the theoretical contradiction pointed out by Reviewer 2. They cannot claim to follow Ingold's relational ontology while also citing cognitive theories that contradict it. They must choose a theoretical lens and apply it consistently throughout the analysis or clearly explain how they are synthesizing these perspectives.

**We have added a theoretical section (2.1) in our literature review that surveys and synthesizes influential case studies and key theoretical framings of animism (e.g., Tylor, Humphrey and Onon, Vitebsky, Bird-David, Viveiros de Castro, Ingold, Descola). While we do not choose just one lens, we do note areas of convergence and difference with these major case studies and models. The primary contiributon of our paper is empirical, because it is a field-based, qualitative study. We also believe it contributes to theory, as would any empirical study, but it is not our main goal to advance a particular theory.

4. Address Specific Suggestions from Both Reviewers:

o Expand the historical context slightly (R1).

**Yes, we added more to the historical context (Section 1 lines 61-74; and section 5.3), from the colonial/missionary period up through contemporary communist rule, and we note the resilience of animistic practices under both systems. However, we note that many historical questions are still considered sensitive, and according to the conditions of our research permit and ethic board approval, we were limited at our field sites to asking questions about Bahnar environmental knowledge.

o Consider alternative terminology to "animism" or justify its use more robustly (R1).

**Yes, we have tried to be clearer and added sections to justify/contextualize the use of “animism”. We do this first in historical context, showing how it has evolved over time in anthropology/ethnography, based on diverse, case studies from locations around the world (Section 2.1). We then show specifically why and how the term applies and how it has been used in the Vietnamese ethnographic context (Section 2.2, 2.3). Finally, we situate the term within a regional (Southeast Asian) context (Section 2.4).

o Add a brief comparative note on Southeast Asian parallels (R1).

**Yes, we added this: Section 2.4 now includes Southeast Asian parallels from Laos, Malaysia, and Cambodia.

o Elaborate on positionality—how the authors' own backgrounds shaped the research (R2).

**Yes, we include a statement on the diverse perspectives and positionalities of the four members of our research team. In response to this comment, we added the additional personal details that we are non-Indigenous, male, Vietnamese (authors #1, #3, #4) and American (author #2). (Section 3.2)

Conclusion:

This manuscript has potential but in its current form, it does not fulfill its ambitious paper. The decision is Major Revision, with the hope that the authors are able to address all comments of reviewer 2 (rejection) points by point. The revisions required are not minor; they are fundamental and concern the paper's core argument, methodological depth, and theoretical coherence. The author's response must convincingly outline how they will address these significant concerns. A resubmitted manuscript will need to be re-evaluated, likely by both original reviewers if possible, to ensure the substantial issues have been resolved.

**We are grateful for the feedback, which has guided and motivated us to make major revisions and improvements.

**We now respond point by point to the reviewers' comments:

Reviewer #1: This is a solid article that is well written, well organized, and generally well-argued. It does appear that the authors have approached their subject with a considerable degree of sensitivity and appropriate cautions with respect to dealing with the population whose spiritual beliefs and practices they are examining. They have appropriately laid out their approach, their practices, and the mechanisms by which they engaged with local collaborators. Thus, it seems that they have taken all of the correct steps in conducting their research.

They do a solid job of giving an overview of Bahnar practices and beliefs and of situating them within the rather complicated historical, religious, and political landscape within which the Bahnar live and operate. Indeed, the pressures of missionary activity, of the modern Catholic Church, and of the contemporary Vietnamese communist state have all impacted the community and its beliefs in various ways and the article makes note of this, which is nice to see.

**We appreciate the positive feedback.

Reviewer #1: While the discussion of “animism” is solid, I would suggest that it is a bit limiting. There is brief acknowledgement that the terms is sometimes viewed as problematic and not used by some scholars, and I think given this, that the authors might have explored alternative terminology. Since “animism” carries a rather significant amount of cultural baggage - related to the kinds of dismissive attitudes of the Vietnamese state regarding ethnic minority populations - the authors might have considered perhaps using terms like “folk religion” or “traditional spiritual beliefs” or something along those lines. I do understand that “animism” captures more specifically the belief regarding the ways in which non-human and even material objects can have animating elements, but I still think the term is a bit loaded.

**Yes, we are aware of some limitations and cultural baggage associated with the term "animism", and while we have considered “folk religion” or “traditional spiritual beliefs” as alternatives, we also find these limiting, as they seem to relegate animism to the sphere of religious beliefs, rather than daily practice based on knowledge. Instead of abandoning the term "animism" we have added discussion (Section 2.1) of how the term has been used and defined differently over time, since it was first introduced in the 19th century, and up to the present day within Anthropology, referring to leading scholars and case studies (e.g., Tylor, Humphrey and Onon, Vitebsky, Bird-David, Viveiros de Castro, Ingold, Descola). We also include examples of how the term has been used and understood by Vietnamese scholars (Section 2.3).

Reviewer #1: Secondly, while there are one or two very brief comparative observations, I guess I’d like to see at least brief attention to situating the Bahnar beliefs within a larger Southeast Asian context (or even just mainland Southeast Asian context). This would reveal the degree to which the Bahnar beliefs, rituals, and practices parallel those found across many ethnic groups and populations across both mainland and island Southeast Asia. Such a comparative comment (at least) would make clear that the Bahnar beliefs/practices are incredibly widespread in the region, even if they are quite different from those practiced by lowland Vietnamese. It would, I think, somehow “mainstream” Bahnar practices as less of an outlier, and more of a very common practice when seen across the broader region.

**Yes, we have now added a new section "2.4 Animism in Southeast Asian context ", which brings in case studies from Malaysia, Cambodia, and Laos. We agree that these case studies help to position Banhar as less of an outlier and more typical of the region, while still having some unique Bahnar characteristics.

Reviewer #1: Finally, while the abstract and the conclusion make mention of how a study of Bahnar practices and beliefs might lead them to ecological preservation and conservation practices, the article barely touches on this, much less actually explores it. In short, while it is possible that their beliefs might translate into preservationist practices, this theory is not explored nor really tested. This is a bit disappointing, because, of course, this is one of the standard claims about groups that are seen as being closer to nature or more in tune with the environment. I’d suggest that the authors make this section of the paper more robust if they are going to make those claims.

**Yes, we agree that this was not a strongly motivated argument, and we have accordingly shifted the focus of the paper to focus on Bahnar animism and knowledge of the environment. We did add several mentions of how Bahnar animism encourages people to care for the forest environment, and also may discourage them from harming or over-exploiting it. We acknowledge that this does not automatically make the Bahnar conservationists, and have therefore omitted our prior claim about "conservation", but we note that animism does seem to introduce an ethos of environmental care (lines 324-327, 872-877, 891-894)

Reviewer #1: Overall, though this is a solid paper on an important ethnic community in Vietnam, and it is presented in an accessible manner that even non-specialists would enjoy and benefit from.

**We thank the reviewer for this positive assessment

Reviewer #2: The article sets out to argue that animist representations of the Bahnar (Central Vietnam) serve to create moral relationships with the non-human environment and thus make Bahnar stewards of the nature they live in. However, the article, while showing that Bahnar everyday life resonates with non-human beings populating the forest, fails to provide evidence for this kind of stewardship. There is no description and analysis of practices that support sustainable use, avoid overuse or needless destruction of species and landscapes. There is also no detailed description how the practices and representations related to those invisible beings called yang regulate moral life among Bahnar – the authors simply mention good or bad behavior without any specification or sense for community morality.

**We agree that this was a weakness of the previous draft, and have accordingly removed the hypothesis or any claim that animism makes the Bahnar stewards of the environment. We acknowledge that animism does not automatically make the Bahnar conservationists, and have deleted our prior claim about "conservation", but we note that animism does seem to introduce an ethos of environmental care (lines 324-327, 872-877, 891-894). We also note that with this paper we hope to contribute to ongoing discussions of how animistic worldviews inform stewardship of nature (lines 187-188), which we think is a more modest and realistic goal.

**We appreciate the feedback and guidance from Reviewer #1, which has motivated us to improve the paper.

Reviewer #2:

1. Concepts

The article focuses a lot on words, and therefore, specific attention should be given to the words the authors use for their analysis.

One of the major problems is the tension between the aim to consider Bahnar cosmology/ontology in its own right and the proficient use of English terms that come with a problematic Eurocentric baggage which the authors do not reflect upon.

**Yes, we have added a section to reflect upon how both French and English terms have been used in an attempt to translate Bahnar concepts.

For instance, they translate the Bahnar c

# Peer review: Decision Letter 1

**DOI:** 10.1371/journal.pone.0354453.r003

**Authors:** Kristiawan Indriyanto

8 Jul 2026

Yang, forest animism, and environmental perception among the Bahnar in the Central Highlands of Vietnam

PONE-D-25-33740R1

Dear Dr. Harrison,

We’re pleased to inform you that your manuscript has been judged scientifically suitable for publication and will be formally accepted for publication once it meets all outstanding technical requirements.

Within one week, you’ll receive an e-mail detailing the required amendments. When these have been addressed, you’ll receive a formal acceptance letter and your manuscript will be scheduled for publication.

An invoice will be generated when your article is formally accepted. Please note, if your institution has a publishing partnership with PLOS and your article meets the relevant criteria, all or part of your publication costs will be covered. Please make sure your user information is up-to-date by logging into Editorial Manager at Editorial Manager® and clicking the ‘Update My Information' link at the top of the page. For questions related to billing, please contact billing support.

If your institution or institutions have a press office, please notify them about your upcoming paper to help maximize its impact. If they’ll be preparing press materials, please inform our press team as soon as possible -- no later than 48 hours after receiving the formal acceptance. Your manuscript will remain under strict press embargo until 2 pm Eastern Time on the date of publication. For more information, please contact onepress@plos.org.

Kind regards,

Kristiawan Indriyanto

Academic Editor

PLOS One

Additional Editor Comments (optional):

I am satisfied that the authors have adequately addressed the reviewers' concerns in this revised version. They have refined the terminology, moderated their claims, and supplied more robust context, all of which have enhanced the clarity and persuasiveness of the work. The manuscript now meets our standards for publication, and I approve it for direct acceptance

Reviewers' comments:

Reviewer's Responses to Questions

**Comments to the Author**

1. If the authors have adequately addressed your comments raised in a previous round of review and you feel that this manuscript is now acceptable for publication, you may indicate that here to bypass the “Comments to the Author” section, enter your conflict of interest statement in the “Confidential to Editor” section, and submit your "Accept" recommendation.

Reviewer #1: All comments have been addressed

Reviewer #3: (No Response)

**********

2. Is the manuscript technically sound, and do the data support the conclusions?

The manuscript must describe a technically sound piece of scientific research with data that supports the conclusions. Experiments must have been conducted rigorously, with appropriate controls, replication, and sample sizes. The conclusions must be drawn appropriately based on the data presented.

Reviewer #1: Yes

Reviewer #3: No

**********

3. Has the statistical analysis been performed appropriately and rigorously?

Reviewer #1: N/A

Reviewer #3: N/A

**********

4. Have the authors made all data underlying the findings in their manuscript fully available?

The PLOS Data policy requires authors to make all data underlying the findings described in their manuscript fully available without restriction, with rare exception (please refer to the Data Availability Statement in the manuscript PDF file). The data should be provided as part of the manuscript or its supporting information, or deposited to a public repository. For example, in addition to summary statistics, the data points behind means, medians and variance measures should be available. If there are restrictions on publicly sharing data—e.g. participant privacy or use of data from a third party—those must be specified.

Reviewer #1: Yes

Reviewer #3: Yes

**********

5. Is the manuscript presented in an intelligible fashion and written in standard English?

PLOS ONE does not copyedit accepted manuscripts, so the language in submitted articles must be clear, correct, and unambiguous. Any typographical or grammatical errors should be corrected at revision, so please note any specific errors here.

Reviewer #1: Yes

Reviewer #3: Yes

**********

6. Review Comments to the Author

Please use the space provided to explain your answers to the questions above. You may also include additional comments for the author, including concerns about dual publication, research ethics, or publication ethics. (Please upload your review as an attachment if it exceeds 20,000 characters)

Reviewer #1: I am impressed by the authors’ responsiveness to the comments and suggestions I offered in my first reading of this manuscript. The new version very effectively addresses my concerns and is a much more robust and convincing work of scholarship. By giving more attention to terminology, narrowing their claims, and offering more robust context, the authors have crafted a better, more nuanced, and more convincing work. This now looks very nice to me and I see no reason it should not proceed directly to publication.

Reviewer #3: I read the revised manuscript before turning to the editor’s and reviewers’ comments on the original submission. I was struck that the concerns that arose for me closely echo those raised in the first round—particularly by Reviewer #2. In this sense, the authors have not succeeded in addressing the main issues identified in the initial review.

To be sure, the authors have responded carefully and in detail to the individual comments. However, in my view, they have largely addressed them at the level of form rather than substance. The core difficulty is a cross-epistemological gap: the manuscript engages cultural-anthropological topics (animism, environmental perception, ritual, and participant observation), yet approaches them in terms more characteristic of other scientific genres—in its use of literature, handling of data, and mode of writing.

As a result, the revisions remain largely additive (more references, more examples, more clarification), while the central issues raised by the reviewers and editor—regarding argument, analytical depth, and theoretical coherence—remain unresolved. An ethnographic case from Vietnam could make a valuable contribution to discussions of

animism and environmental perception. However, addressing these issues would require not further revision but a fundamental reworking of the paper’s analytical and stylistic approach.

For these reasons, I recommend rejection. It may be that the manuscript, in a form closer to its present one, would be better suited to a journal with different disciplinary expectations.

**********

7. PLOS authors have the option to publish the peer review history of their article (what does this mean?). If published, this will include your full peer review and any attached files.

If you choose “no”, your identity will remain anonymous but your review may still be made public.

**Do you want your identity to be public for this peer review?**  For information about this choice, including consent withdrawal, please see our Privacy Policy.

Reviewer #1: No

Reviewer #3: No

**********

# Editor report: Acceptance letter

**DOI:** 10.1371/journal.pone.0354453.r004

**Authors:** Kristiawan Indriyanto

PONE-D-25-33740R1

PLOS One

Dear Dr. Harrison,

I'm pleased to inform you that your manuscript has been deemed suitable for publication in PLOS One. Congratulations! Your manuscript is now being handed over to our production team.

At this stage, our production department will prepare your paper for publication. This includes ensuring the following:

* All references, tables, and figures are properly cited

* All relevant supporting information is included in the manuscript submission,

* There are no issues that prevent the paper from being properly typeset

You will receive further instructions from the production team, including instructions on how to review your proof when it is ready. Please keep in mind that we are working through a large volume of accepted articles, so please give us a few days to review your paper and let you know the next and final steps.

Lastly, if your institution or institutions have a press office, please let them know about your upcoming paper now to help maximize its impact. If they'll be preparing press materials, please inform our press team within the next 48 hours. Your manuscript will remain under strict press embargo until 2 pm Eastern Time on the date of publication. For more information, please contact onepress@plos.org.

You will receive an invoice from PLOS for your publication fee after your manuscript has reached the completed accept phase. If you receive an email requesting payment before acceptance or for any other service, this may be a phishing scheme. Learn how to identify phishing emails and protect your accounts at https://explore.plos.org/phishing.

If we can help with anything else, please email us at customercare@plos.org.

Thank you for submitting your work to PLOS ONE and supporting open access.

Kind regards,

PLOS ONE Editorial Office Staff

on behalf of

Dr. Kristiawan Indriyanto

Academic Editor

PLOS One
